# Dual-Isotope SPECT/CT Imaging of the Tuberculosis Subunit Vaccine H56/CAF01: Induction of Strong Systemic and Mucosal IgA and T-Cell Responses in Mice Upon Subcutaneous Prime and Intrapulmonary Boost Immunization

**DOI:** 10.3389/fimmu.2018.02825

**Published:** 2018-11-30

**Authors:** Aneesh Thakur, Cristina Rodríguez-Rodríguez, Katayoun Saatchi, Fabrice Rose, Tullio Esposito, Zeynab Nosrati, Peter Andersen, Dennis Christensen, Urs O. Häfeli, Camilla Foged

**Affiliations:** ^1^Department of Pharmacy, Faculty of Health and Medical Sciences, University of Copenhagen, Copenhagen, Denmark; ^2^Faculty of Pharmaceutical Sciences, The University of British Columbia, Vancouver, BC, Canada; ^3^Department of Physics and Astronomy, The University of British Columbia, Vancouver, BC, Canada; ^4^Department of Infectious Disease Immunology, Statens Serum Institut, Copenhagen, Denmark

**Keywords:** H56/CAF01 vaccine, SPECT/CT imaging, dual-isotope ^111^In/^67^Ga, pulmonary immunization, T cells, mucosal immunity, nanomedicine, drug delivery

## Abstract

Pulmonary tuberculosis (TB), which is caused by *Mycobacterium tuberculosis* (*Mtb)*, remains a global pandemic, despite the widespread use of the parenteral live attenuated Bacillus Calmette–Guérin (BCG) vaccine during the past decades. Mucosal administration of next generation TB vaccines has great potential, but developing a safe and efficacious mucosal vaccine is challenging. Hence, understanding the *in vivo* biodistribution and pharmacokinetics of mucosal vaccines is essential for shaping the desired immune response and for optimal spatiotemporal targeting of the appropriate effector cells in the lungs. A subunit vaccine consisting of the fusion antigen H56 (Ag85B-ESAT-6-Rv2660) and the liposome-based cationic adjuvant formulation (CAF01) confers efficient protection in preclinical animal models. In this study, we devise a novel immunization strategy for the H56/CAF01 vaccine, which comply with the intrapulmonary (i.pulmon.) route of immunization. We also describe a novel dual-isotope (^111^In/^67^Ga) radiolabeling approach, which enables simultaneous non-invasive and longitudinal SPECT/CT imaging and quantification of H56 and CAF01 upon parenteral prime and/or i.pulmon. boost immunization. Our results demonstrate that the vaccine is distributed evenly in the lungs, and there are pronounced differences in the pharmacokinetics of H56 and CAF01. We provide convincing evidence that the H56/CAF01 vaccine is not only well-tolerated when administered to the respiratory tract, but it also induces strong lung mucosal and systemic IgA and polyfunctional Th1 and Th17 responses after parenteral prime and i.pulmon. boost immunization. The study furthermore evaluate the application of SPECT/CT imaging for the investigation of vaccine biodistribution after parenteral and i.pulmon. immunization of mice.

## Introduction

*Mycobacterium tuberculosis* (*Mtb*), which is causing pulmonary tuberculosis (TB), has infected humans for thousands of years and it is estimated that about one third of the global population is latently infected with TB, which continues to infect 10 million and kill more than 1.5 million people every year (1–3). Increased multi-drug resistance and extensive drug-resistance against existing antibiotics, and the very slow progress in developing new types of antibiotics might result in even poorer prognosis in the future if novel solutions to fight TB are not found. To date, the only licensed TB vaccine remains the live attenuated Bacillus Calmette–Guérin (BCG) vaccine developed from the closely related *Mycobacterium bovis*, causing TB in cattle. The BCG vaccine is one of the most widely used vaccines ever in the world. It is effective against disseminated childhood TB, but it fails to control pulmonary TB in adolescents and adults (4, 5). Hence, there is an urgent medical need for designing novel vaccines and delivery strategies, which can effectively boost BCG-primed immune responses in adolescents and adults, and ultimately induce protective immunity against TB (6, 7).

After infection in the lungs, *Mtb* adopts a variety of immune evasion strategies, which chiefly includes suppression of an innate immune response and subsequently delaying T cell responses in the lungs by approximately 2 weeks (8). These evasion strategies enable *Mtb* to proliferate in the lungs (8–10), eventually explaining the poor efficacy of parenteral BCG vaccination in humans (8, 11). Therefore, homologous or heterologous boost immunization strategies aiming at inducing T-cell immunity in the lungs have the potential to fill this gap (6, 10, 12). Recent preclinical studies have reported induction of protective T-cell immunity in the lungs upon mucosal vaccination *via* the airways (13–18). Mucosal immunization in the lungs has been shown to activate local dendritic cells (DCs) (19) to induce antigen-specific T cells, which effectively home back to the lung parenchyma, where they control initial *Mtb* replication after infection (6, 18). However, almost all TB vaccine candidates in the global clinical pipeline are administered parenterally (20). Subunit vaccines based on adjuvanted, recombinant TB proteins represent an attractive approach for airway mucosal vaccination (21–23). Besides, vaccine delivery in lungs through inhalation may circumvent the potential safety concerns associated with administration of gene delivery systems, live attenuated organisms, and potentially neurotoxic adjuvant molecules through the nasal route (24, 25). However, thorough safety assessment of airway mucosal vaccination is required.

Understanding the biodistribution and pharmacokinetics of injectable and mucosally administered subunit vaccines is essential (i) for shaping and orchestrating the desired immune response and (ii) for optimal spatiotemporal targeting of the appropriate populations and numbers of effector cells at the site of infection in the lungs. Molecular imaging assessment of such low-dose biological medicinal products using for example single-photon emission computerized tomography (SPECT), allows for the characterization and quantification of biological processes at the cellular and subcellular level in intact living subjects with sufficient spatial and temporal resolution (26). SPECT imaging is based on the measurement of single photons emitted by γ-emitting radionuclides, e.g., ^99m^Technitium, ^111^Indium (^111^In), and ^67^Gallium (^67^Ga). Furthermore, SPECT imaging is non-invasive and quantitative, permitting uniform and repeated measurements using a single animal subject, thus exploiting the statistical power of longitudinal studies and reducing the required number of animals. In addition, it allows for tracer multiplexing, where several isotopes of different energies can be used in the same animal. Hence, this imaging modality is an effective substitute for conventional *ex vivo* biodistribution studies, which usually require a larger number of animals assessed at multiple time points. In addition, high structural resolution can be achieved by combining the robustness of morphological/anatomical [e.g., computer tomography (CT)] and molecular imaging modalities, which is referred to as multimodality imaging, such as SPECT/CT (26–28). SPECT/CT imaging has been successfully applied in many areas of medical science, but very few reports have been published on SPECT/CT imaging-based investigations for vaccines.

The TB protein subunit vaccine H56/CAF01, which comprises the multi-stage subunit TB fusion protein H56 (Ag85B-ESAT-6-Rv2660c) co-formulated with the liposomal adjuvant referred to as cationic adjuvant formulation 01 (CAF01), has been shown to induce protective immunity before and after *Mtb* exposure in preclinical models (29, 30). H56 is currently tested in a clinical phase 2a trial with the IC31® (Valneva, Lyon, France) adjuvant (31). CAF01, which is based on the surfactant dimethyldioctadecylammonium (DDA) bromide and the glycolipid trehalose-6,6′-dibehenate (TDB), has been shown to deliver antigen to and activate DCs through the Toll-like receptor (TLR)-independent Syk-CARD9 pathway (32), and it induces a Th1- and Th17-biased CD4^+^ T cell response along with a humoral immune response (33, 34). In clinical phase I trials, CAF01 has been found safe, well-tolerated and immunogenic when co-administered with a protein-based TB antigen (NCT00922363) or a cocktail of HIV-1 peptides (NCT01141205). In preclinical studies in mice, CAF01 mixed with H56 has been shown to be safe and immunogenic following intranasal prime and/or boost immunizations (22). Thus, H56/CAF01 is a safe and efficacious multi-faceted TB vaccine.

In this study, we tested for the first time mucosal application of H56/CAF01 using intrapulmonary (i.pulmon.) administration in the airways. We also report the first radiolabeling and preclinical SPECT/CT imaging of the biodistribution and pharmacokinetics of a subunit vaccine. We provide compelling evidence that mucosal administration of H56/CAF01 in the airways induces high levels of antigen-specific lung mucosal IgA and polyfunctional CD4^+^ T-cell responses following intramuscular (i.m.) priming and i.pulmon. mucosal boost immunization. In addition, strong systemic IgA and polyfunctional CD4^+^ T-cell responses are induced, which are comparable to the systemic responses induced upon homologous i.m. prime-boost immunization. We show successful dual-isotope radiolabeling of H56 and CAF01 with ^111^In and ^67^Ga and observe pronounced differences in the pharmacokinetics of H56 and CAF01 following i.pulmon. immunization. Hence, this study underlines the promising potential of H56/CAF01 as a vaccine candidate for airway mucosal immunization, and thus provides the basis for its further preclinical and clinical development as an inhalable and self-administrable aerosol vaccine.

## Materials and methods

### Materials

DDA and 18:0 1,2-distearoyl-*sn*-glycero-3-phosphoethanolamine-N-diethylenetriaminepentaacetic acid (ammonium salt) (PE-DTPA) were obtained from Avanti Polar Lipids (Alabaster, AL, USA), and TDB was purchased from Niels Clauson-Kaas A/S (Farum, Denmark). S-2-(4-Isothiocyanatobenzyl)-diethylenetriamine pentaacetic acid (p-SCN-Bn-DTPA) and 2-S-(4-Isothiocyanatobenzyl)-1,4,7-triazacyclononane-1,4,7-triacetic acid (p-SCN-Bn-NOTA) were procured from Macrocyclics (Plano, TX, USA). Recombinant H56 protein was produced in *E. coli* as previously described (35). It was reconstituted in 20 mM glycine buffer (pH 8.8), checked for purity and validated for residual DNA, endotoxins and bioburden following internal good manufacturing practice standards. All other chemicals and reagents were of analytical grade and were acquired from commercial suppliers.

### Preparation and physicochemical characterization of vaccine formulations

Liposomes were prepared by using the thin film method and characterized for average intensity-weighted hydrodynamic diameter (*z*-average), polydispersity index (PDI), and zeta-potential using a Malvern Zetasizer Nano-ZS (Malvern Instruments, Worcestershire, UK) by dynamic light scattering using photon correlation spectroscopy and Laser-Doppler electrophoresis, respectively, as previously described (33). Briefly, weighed amounts of DDA and TDB (5:1, w/w) were dissolved in chloroform/methanol (9:1, v/v) in a round bottom flask. The organic solvents were removed by rotary evaporation under vacuum resulting in the formation of a thin lipid film. The lipid film was washed twice with 99% (v/v) ethanol and dried overnight under vacuum to remove trace amounts of the organic solvents. On the following day, the lipid film was hydrated with 10 mM Tris buffer (pH 7.4), sonicated for 5 min using an ultrasound cleaner (Branson Ultrasonic Cleaner, Danburry, CT, USA), and heated to 60°C for 1 h in a water bath with vortexing every 10th min. In addition, the liposome dispersions were tip-sonicated 20 min after the rehydration for 20 s with a 150 W Branson tip-sonicator to reduce the particle size. The final concentration of CAF01 was 20/4 mg/mL of DDA/TDB, corresponding to a molar ratio of 89:11. The H56 solution was mixed with equal volumes of CAF01 liposome dispersions at concentrations of 5 and 10 μg/mL, respectively, and H56 was allowed to adsorb to CAF01 by incubation for 30 min at room temperature (36).

### Radiolabeling of CAF01 and H56

For radiolabeling of CAF01 liposomes, DDA and TDB were dissolved in chloroform/methanol (9:1, v/v) with 18:0 PE-DTPA (10%, w/w), and the liposome dispersions were prepared in 100 mM HEPES buffer (pH 7.0) as described above. All H56/CAF01 vaccine formulations were radiolabeled as follows: The 18:0 PE-DTPA (10% w/w) chelated CAF01 liposomes were purified using 10 kDa centrifugal filters (Amicon® Ultra 0.5 mL, Merck Life Science, Hellerup, Denmark) to remove excess chelator. For ^111^In-labeling, ^111^InCl_3_ (55.5 MBq, 5 μL in 0.1 M HCl) was added to purified DTPA-CAF01 in HEPES buffer (175 μL, 100 mM, pH 7.0), and the reaction mixture was stirred (600 rpm) at room temperature for 1 h. Instant thin layer chromatography (ITLC) showed high labeling efficiency, and ^111^In-CAF01 was therefore used without further purification. The collected ^111^In-CAF01 dispersion was diluted to a final volume of 275 μL with HEPES buffer, and non-labeled H56 (75 μL, 1 μg/μL) was allowed to adsorb for 30 min to the liposomes before administration.

The H56 protein in 20 mM glycine buffer (pH 8.8) was buffer-exchanged into 100 mM sodium bicarbonate buffer (pH 8.3) using 10 kDa ultracentrifugal filters (Amicon® Ultra 0.5 mL, Merck Life Science) and incubated on an Eppendorf shaker for 5 h at 10°C with p-SCN-Bn-DTPA or p-SCN-Bn-NOTA, respectively, at a molar ratio of 1:5. After 5 h, unreacted DTPA or NOTA was removed by centrifugation through 30 kDa centrifugal filters (Amicon® Ultra 0.5 mL, Millipore, Ontario, Canada), washed and buffer-exchanged into 100 mM HEPES buffer (pH 7.0). For ^111^In-labeling, ^111^InCl_3_ (148 MBq/200 μg of H56) was added to H56, and the mixture was incubated for 1 h on an Eppendorf shaker at room temperature. For ^67^Ga-labeling, ^67^GaCl_3_ (19.5 MBq/70 μg of H56) was added to H56 and incubated for 1 h on an Eppendorf shaker at room temperature. ^111^In-H56 or ^67^Ga-H56 was diluted to 500 or 350 μL, respectively, with HEPES buffer and mixed with non-labeled or ^111^In-CAF01 liposomes 30 min prior to immunization.

### Radiolabeling efficiency and purity of ^111^In-H56, ^67^Ga-H56 and ^111^In-CAF01

The radiochemical purity and labeling efficiency were measured by ITLC using a Tec-Control stationary phase (Biodex Medical Systems, Shirley, NY, USA) and a 0.1 M EDTA (pH 4) mobile phase. The ^111^In-CAF01 and ^111^In-H56 or ^67^Ga-H56 complexes, which have larger molecular weight, would remain at the origin, while the free ^111^In elutes with the mobile phase at the solvent front [Retention Factor (Rf) (^111^In-CAF01, ^111^In-H56, or ^67^Ga-H56) = 0, Rf (free ^111^In^3+^ or free ^67^Ga^3+^) = 1]. The location of the radioactivity was assessed using a Cyclone Phosphorimager and a photostimulable phosphor plate (Perkin Elmer, Waltham, MA, USA). A NanoDrop spectrophotometer (Thermo Fischer Scientific, Waltham, MA, USA) was used to assess the protein concentration [A_280_ with E1% set to 20.5 L/(g·cm)] and determine the specific activity. ^111^In-H56 was analyzed further *via* 10% native- and SDS-PAGE using established protocols in the lab.

### *In vivo* SPECT/CT imaging

The imaging studies, which were conducted at The University of British Columbia, were performed in accordance with the Canadian Council on Animal Care (CCAC), and the protocols were approved by the Animal Care Committee (ACC) of the University of British Columbia (A16-0150). Five-to 6-week old healthy female C57BL/6 mice were purchased from Charles River and allowed free access to food and water. In the first study, mice were allocated into four groups of three individuals. All groups were immunized s.c. (at the base of the tail toward the right) or i.pulmon. with either 10 μg cold H56 adjuvanted with ^111^In-CAF01 (125/25 μg DDA/TDB) or 10 μg ^111^In-H56 adjuvanted with cold CAF01 (125/25 μg DDA/TDB) in a total volume of 200 μL or 50 μL, respectively. In the prime-boost immunization study, mice were distributed into two groups of three. Two groups were primed s.c. with 200 μL of either 5 μg cold, unadjuvanted H56 or 5 μg cold H56 adjuvanted with cold CAF01 (250/50 μg DDA/TDB). Two weeks later, booster immunization was performed by i.pulmon. administration of either 10 μg unadjuvanted ^67^Ga-H56 or ^67^Ga-H56 adjuvanted with ^111^In-CAF01 (125/25 μg DDA/TDB) in a total volume of 50 μL. The i.pulmon. administration was performed using a MicroSprayer®/Syringe Assembly (MSA-250-M, Penn-Century, Inc., Wyndmoor, PA, USA) according to a previously reported method (37). In brief, mice were anesthetized by intraperitoneal (i.p.) injection of Ketamine (Ketamin, MSD Animal Health, Havneholmen, Denmark)/Xylazine (Rompun Vet, Bayer, Copenhagen, Denmark) 100/5 mg/kg, respectively, and placed on a rodent tilting work stand at a 45° angle by the upper incisors (Hallowell EMC, Pittsfield, MA, USA). For the prime-boost immunization study, anesthesia was induced with 5% isoflurane, the mice were placed on a rodent tilting intubation stand with an integrated anesthesia facemask (Kent Scientific, Torrington, CT, USA), and anesthesia was maintained with 3% isoflurane. A cold light source with a flexible fiber-optics arm (SCHOTT AG, Mainz, Germany) was used for optimal illumination of the trachea, which appeared as a white light spot. A cotton swab was used to open the lower jaw of the mouse, and the tongue was displaced to the left with a blunted forceps. A laryngoscope (WelchAllen, NY, USA) fitted with a 41 mm intubation specula (Halowell EMC) was used with the other hand for maximal oropharyngeal exposure. After a clear view of the trachea, the laryngoscope with the specula was taken out, and 50 μL of the formulation was administered intratracheally with the MicroSprayer®/Syringe Assembly right above the carina (first bifurcation) to ensure uniform delivery into both lungs. The tip of the syringe was immediately withdrawn, and the mouse was taken off the intubation stand. During SPECT/CT imaging, the mice were anesthetized using isoflurane (1–3% for maintenance, up to 5% for induction) and oxygen from a precision vaporizer, and they received s.c. injection of Lactated Ringer's solution (0.5 mL, B. Braun, Mississauga, Canada) for hydration prior to the SPECT/CT imaging scan. The SPECT/CT imaging was performed using a VECTor/CT preclinical small animal scanner (MILabs, Utrecht, The Netherlands). The respiratory rate and body temperature of the mice were monitored continuously during the scans, and the isoflurane dose and animal anesthesia bed temperature were adjusted accordingly. All animals recovered after each scan. Mice were euthanized 144 h post-administration using CO_2_, the blood was collected by cardiac puncture, and the tissues were isolated to quantify the biodistribution.

### SPECT/CT parameters and image reconstruction

Whole-body SPECT ^111^In and ^67^Ga data were acquired using an integrated VECTor/CT preclinical scanner (MILabs) equipped with an XUHS-2 mm mouse multi-pinhole collimator. Dynamic whole-body scans were acquired in list-mode format over 40 min (10 min/frame) post-s.c. or -i.pulmon. administration to study the biodistribution of the protein and the liposomes every 10 min. Subsequently, static 40 min scans were performed for the 6 and 24 h scans, while longer imaging times of 60 and 90 min were performed at subsequent imaging time-points (96 and 144 h) after vaccine administration to increase the statistical signal and the quality of the ^111^In and ^67^Ga images. Following each SPECT acquisition, a whole-body CT scan was acquired to obtain anatomical information, and both images were registered. For the first imaging study, the ^111^In photopeak window was centered at 171 keV with a 20% energy window width. For assessment of the biodistribution in the prime-boost immunization study, the ^111^In photopeak window was centered at 20 keV with a 60% energy window width, while the ^67^Ga photopeak was centered at 96 keV with a 20% energy window width. For quantitative analysis, SPECT image reconstructions were carried out using the pixel-ordered subset expectation maximization (POSEM) algorithm (38), which includes resolution recovery and compensation for distance-dependent pinhole sensitivity. Further details are provided in the Supplementary Section.

### Biodistribution

After the last scan at day 6 (144 h), blood samples were collected by cardiac puncture, and a complete biodistribution assessment was conducted by collecting heart, liver, kidneys, lungs, lymph nodes (LNs), small intestine, bladder, muscle, site of injection, spleen, stomach, bone, trachea, and pancreas. The organs were cleaned from blood and weighed, and the radioactivity was measured using a gamma counter (Packard Cobra II autogamma counter, Perkin Elmer, Waltham, MA, USA). The calibration factor for ^111^In was 163631 cpm and ^67^Ga was 78395 cpm (instrument-specific). The total weight of the organs was used to calculate the administered dose per organ (%AD/organ).

### Immunizations

Six-to 8-week old female CB6F1 (BALB/c x C57BL/6, Scanbur, Karlslunde, Denmark) hybrid mice were acquired and acclimatized for 1 week before experimental manipulation. All experimental work related to vaccine immunogenicity was performed at University of Copenhagen and approved by the Danish National Experiment Inspectorate under permit 2016-15-0201-01026. The studies were performed in accordance with the European Community directive 86/609 for the care and use of laboratory animals. Mice were assigned to five groups of six individuals, and they were immunized three times using a dose volume of 50 μL of Tris buffer (pH 7.4) at 2-week intervals, which is in line with our previous studies showing that three immunizations are required when applying the s.c. route of administration for optimal immune stimulation (29, 39). The immune responses were evaluated 2 weeks after the final immunization. All vaccine priming was performed by i.m. administration in the right thigh muscles, while the two booster immunizations were given i.pulmon. The first group of mice was primed with saline (*n* = 3) and 5 μg unadjuvanted H56 (*n* = 3), respectively, and boosted i.pulmon. with 10 μg unadjuvanted H56. The second group (250/50 3^*^i.m., *n* = 6) was primed and boosted i.m. with 5 μg H56 adjuvanted with CAF01 at a dose of 250/50 μg DDA/TDB. Groups 3–5 (*n* = 6 each) were all primed i.m. with 5 μg H56 adjuvanted with CAF01 at a dose of 250/50 μg DDA/TDB. These three groups were boosted i.pulmon. with 10 μg H56 adjuvanted with CAF01 at a dose of 125/25 (125/25 i.m./2^*^i.pulmon.) and 250/50 (250/50 i.m./2^*^i.pulmon.) for groups 3 and 4, respectively, and 500/100 μg DDA/TDB (500/100 i.m./2^*^i.pulmon.) for group 5. CAF01 alone does not induce immunological responses (40, 41). Hence, it was not included as a control in this study. In addition, we have previously shown that there is no difference between s.c. (29) and i.m. (42) immunization with the H56/CAF01 vaccine in mice with respect to immunogenicity and protection against TB infection. Hence, we chose the i.m. route of administration for evaluation of vaccine immunogenicity.

### *In vivo* staining

Anti-CD45.2 FITC (clone 104; BD Biosciences, Lyngby, Denmark) was diluted to 10 μg/mL in sterile PBS, and 250 μL of the diluted antibody was injected (i.v.) *via* the tail vein 3 min prior to euthanasia of the CB6F1 mice.

### Sample collection and cell preparation

Blood samples were taken by cardiac puncture. Serum was isolated by allowing the blood to clot at room temperature, and the clot was removed by centrifugation at 2,000 × g for 10 min. Subsequently, serum was collected and stored at −20°C. The lungs were aseptically removed from the euthanized mice and transferred to gentleMACS C tubes (Miltenyi Biotec Norden AB, Lund, Sweden) containing 2 mL of RPMI 1640 (Sigma-Aldrich, Brøndby, Denmark), 5% (v/v) FCS (Gibco Thermo Fisher, Hvidovre, Denmark) and 0.8 mg/mL collagenase type IV (Sigma-Aldrich, St. Louis, MO, USA). They were dissociated into 1-2 mm sized pieces using the gentleMACS dissociator (Miltenyi Biotec Norden AB). After 1 h incubation at 37°C, the lung pieces were dissociated again using the gentleMACS dissociator and centrifuged at 700 × g for 5 min. The lung supernatants were collected and stored at −20°C until antibody detection. The lung cell pellets were homogenized using a cell strainer (Falcon, Durham, NC, USA) and washed twice using RPMI-1640 (Sigma-Aldrich). The spleen, the lung-draining tracheobronchial and mediastinal lymph nodes (TLNs and MLNs) and lymph nodes draining the site of i.m. injection, i.e., inguinal (ILNs) and popliteal lymph nodes (PLNs), were aseptically collected. Single-cell suspensions were obtained from the spleens and draining LNs by homogenizing the organs through a nylon mesh cell-strainer (Falcon) followed by two washings with RPMI 1640. The cells were grown in microtiter plates (Nunc, Roskilde, Denmark) containing 2 × 10^5^ cells per well for cytokine assays, or 1 × 10^6^ cells per well for flow cytometry in 100 μL RPMI-1640 (Sigma-Aldrich) supplemented with 5 × 10^−5^ M 2-mercaptoethanol (Gibco Thermo Fisher), 1% (v/v) sodium pyruvate (Sigma-Aldrich), 1% (v/v) penicillin-streptomycin (Gibco Thermo Fisher), 1% HEPES (Gibco Thermo Fisher), and 10% (v/v) FCS (Gibco Thermo Fisher).

### Antibody detection

Maxisorp™ plates (Nunc) were coated with 0.5 μg/mL of H56 solution. Serum and lung supernatants were 5-fold or 10-fold serially diluted 8-12 times from a 1:1 dilution with bicarbonate buffer. IgA, IgG1, IgG2a, IgG2b, IgG2c, and IgM were detected with HRP-conjugated secondary antibodies (Supplementary Table S1). 3,3′,5,5′-tetramethylbenzidine (TMB) Plus2 (Kem-En-Tec, Taastrup, Denmark) was used as substrate. Non-linear regression analysis was performed on serum O.D. values to calculate the ELISA mid-point titers, i.e., EC_50_ as previously described (43).

### Cytokine assays

For the IFN-γ and IL-17A assays, lung and spleen cells were stimulated with 2 μg/mL H56 antigen. Wells containing medium alone or 5 μg/mL concanavalin A (Sigma-Aldrich) were included as negative and positive controls, respectively. The supernatants were harvested after 72 h incubation at 37°C/5% CO_2_, and the IFN-γ and IL-17A production were quantified by using a standard ELISA protocol. Briefly, purified rat anti-mouse IFN-γ and IL-17A (Biolegend, San Diego, CA, USA) were used as capture antibodies, and biotin-conjugated rat anti-mouse IFN-γ and IL-17A (Biolegend) were used as detection antibodies, followed by HRP-conjugated streptavidin (BD Biosciences, Kongens Lyngby, Denmark) and TMB Plus2 ready-to-use substrate (Kem-En-Tec). The enzymatic reaction was stopped at optimal color development with 0.2 M H_2_SO_4_, and the absorbance was read at a wavelength of 450 nm.

### Flow cytometry

The isolated lung, spleen and lymph node cells were stimulated *in vitro* in media containing 1 μg/mL anti-CD28 (37.51; BD Biosciences) and 1 μg/mL anti-CD49d (9C10; BD Biosciences) without antigen, or in the presence of 2 μg/mL H56 for 1 h, followed by incubation for 5 h at 37°C in the presence of 10 μg/mL brefeldin A (Sigma-Aldrich) and 0.7 μL/mL monensin/Golgi-stop (BD Biosciences). Following overnight storage at 4°C, the cells were washed with FACS buffer [PBS containing 0.1% (w/v) sodium azide and 1% (v/v) FCS] and stained for 30 min at 4°C for surface markers using anti-CD4-APC-eF780 (RM4-5; eBioscience, San Diego, CA, USA) and anti-CD44-APC (IM7; Biolegend) mAbs. For intracellular staining, cells were washed with FACS buffer, fixed and permeabilized using the Cytofix/Cytoperm kit (BD Biosciences) and stained for 30 min at 4°C for intracellular cytokines using anti-IFN-γ-PE-Cy7 (XMG1.2; eBioscience), anti-TNF-α-PE (MP6-XT22; eBioscience), and anti-IL-17A-PerCP-Cy5.5 (eBio17B7; eBioscience). Cells were twice washed, resuspended in FACS buffer and analyzed using an LSRFortessa flow cytometer (BD Biosciences). Gates for the surface markers are based on fluorescence-minus-one controls. All flow cytometric analyses were performed using the FlowJo software v10 (Tree Star, Ashland, OR, USA).

### Statistical analysis

GraphPad Prism software (Graphpad Software Inc, La Jolla, CA, USA) was used to perform all statistical analyses. SPECT/CT imaging-based SUVs and *ex vivo* biodistributions were compared between the groups by two-way ANOVA and multiple comparisons were performed by Sidak's post-test. Immune responses were compared between the groups by ANOVA (IFN-γ and IL-17 responses, and antibody responses measured by ELISA) or two-way ANOVA (polyfunctional T cells) at a 0.05 significance level, and pair-wise comparison was performed using Tukey's post-test. A value of *p* < 0.05 was considered significant.

## Results

### Radiolabeling of CAF01 liposomes and H56 protein does not influence their physicochemical characteristics

**Figure 1:**
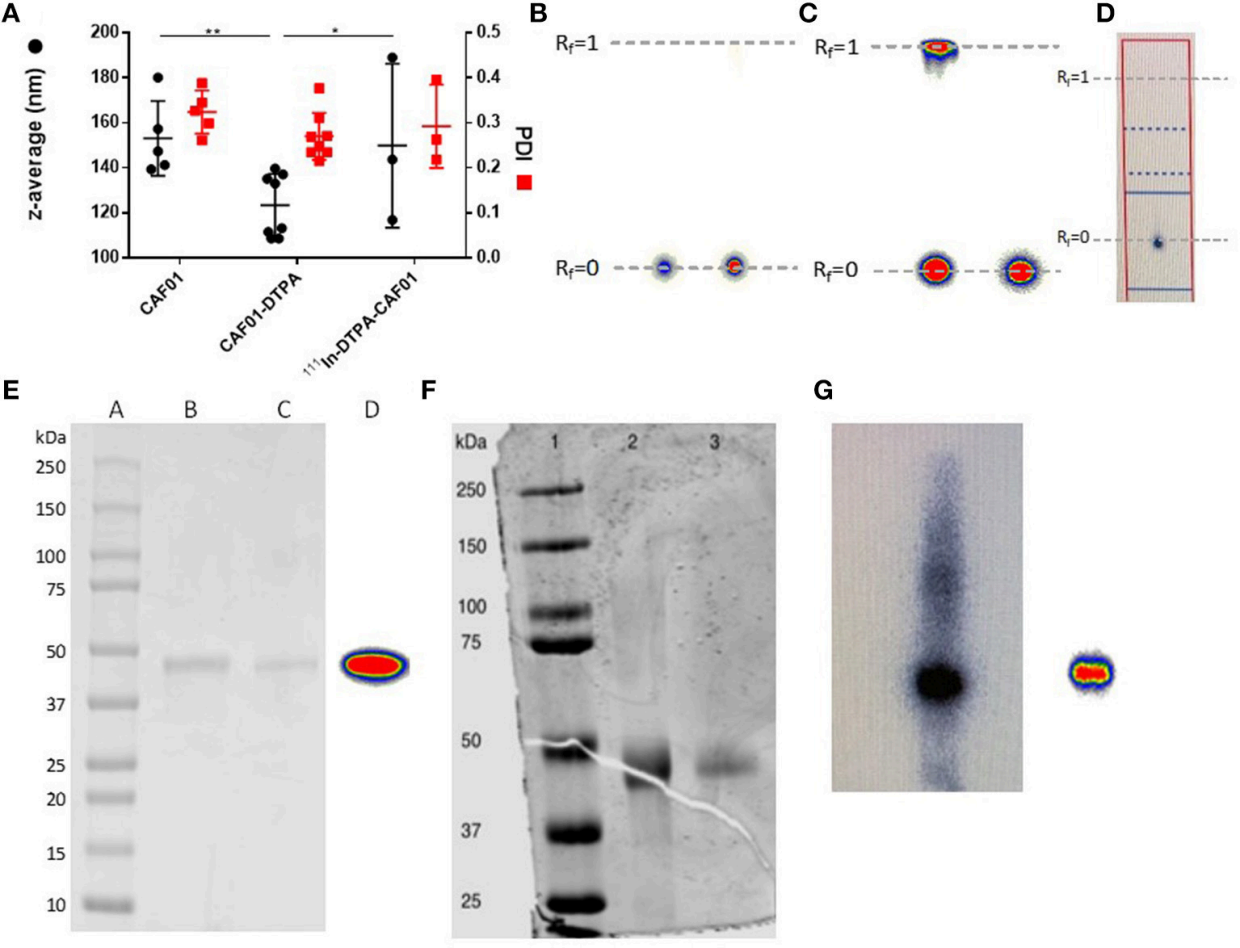
Physicochemical properties of radiolabeled CAF01 liposomes and H56 protein. **(A)** Chelation of DTPA and radiolabeling of CAF01 with ^111^In do not significantly influence the average intensity-weighted hydrodynamic diameter (*z*-average, circles, left axis) and polydispersity index (PDI) (squares, right axis), respectively. Statistical analysis: two-way ANOVA and Tukey's post-test. Bars represent mean values ± s.d., *n* = 5 (CAF01), *n* = 8 (CAF01-DTPA), and *n* = 3 (^111^In-DTPA-CAF01). *p < 0.05 and **p < 0.001. **(B)** Instant thin layer chromatography (ITLC) analysis of ^111^In-DTPA-CAF01. Free ^111^In^3+^ migrates with the solvent front (R_f_ = 1.0), whereas ^111^In-DTPA-CAF01 stays at the origin (R_f_ = 0). Left is ^111^In-DTPA-CAF01 for s.c. injection and right is ^111^In-DTPA-CAF01 for i.pulmon. administration. **(C)** ITLC analysis of ^111^In-DTPA-H56, where free ^111^In^3+^ migrates with the solvent front (R_f_ = 1.0, left) and ^111^In-DTPA-H56 stays at the origin (R_f_ = 0, right). **(D)** ITLC analysis of ^67^Ga-NOTA-H56, where ^67^Ga-NOTA-H56 stays at the origin (R_f_ = 0). **(E)** SDS-PAGE analysis of ^111^In-labeled H56 protein. Lane A represents a protein ladder, lane B is unmodified H56 protein, lane C is ^111^In-DTPA-H56 protein, and lane D is ^111^In-DTPA-H56 protein showing the phosphorimager radioactivity signal. **(F)** SDS-PAGE analysis of ^67^Ga-labeled H56 protein. Lane 1 represents the protein ladder, lane 2 is NOTA-H56, and lane 3 is ^67^Ga-NOTA-H56 protein. **(G)** Phosphorimager radioactivity signal from ^67^Ga-NOTA-H56 protein.

CAF01 was prepared by using the thin film method combined with ultrasonication as previously described (44). The resulting unilamellar liposomes had an average hydrodynamic diameter of approximately 153 ± 17 nm, a PDI of 0.324 ± 0.048 (Figure 1A), and a zeta-potential of 63.6 ± 11.7 (*n* = 5, results not shown), which are in accordance with previously reported values for CAF01 prepared using the same method (44–46). The chelation with 18:0 PE-DTPA significantly decreased the size of the CAF01 liposomes (average size = 123 ± 11 nm, PDI = 0.270 ± 0.021, Figure 1A, zeta-potential = 69.9 ± 8.2, *n* = 8, results not shown). However, the subsequent radiolabeling with ^111^In (average size = 149.8 ± 36 nm, PDI = 0.292 ± 0.092, Figure 1A, zeta-potential = 55.8 ± 8.52, *n* = 3, results not shown) had no effect on the physicochemical properties of CAF01 liposome dispersions as their hydrodynamic diameter, PDI and zeta-potential are maintained after radiolabeling (Figure 1A). The radiochemical purity and labeling efficiency of ^111^In-CAF01 (Figure 1B), ^111^In-H56 (Figure 1C) and ^67^Ga-H56 (Figure 1D) were measured using ITLC. ^111^In-labeling of both CAF01 and H56 using the 18:0 PE-DTPA and p-SCN-Bn-DTPA chelator, respectively, was consistently accomplished with labeling efficiencies of approximately 95% for ^111^In-CAF01 (Figure 1B) and 80% for ^67^Ga-H56 (Figure 1C). The average number of bound DTPA molecules was 5 per molecule lipid molecule in CAF01 and 1 per molecule of H56, and the average number of bound NOTA molecules was 1 per molecule of H56. ^67^Ga-labeling of H56 using the p-SCN-Bn-NOTA chelator resulted in labeling efficiencies of ~93% (Figure 1D). The protein concentrations of the radiolabeled ^111^In-H56 and ^67^Ga-H56 were 1 and 0.9 mg/mL, respectively. Both ^111^In- (Figure 1E) and ^67^Ga-labeled H56 protein (Figures 1F,G) displayed their original molecular weight of 48 kDa (36) determined using SDS-PAGE, suggesting that the radiolabeling procedure did not cause any major modification of the overall size of H56. These data suggest that radiolabeling does not affect the physicochemical properties of CAF01 and H56.

### The H56/CAF01 vaccine remains in the lungs following i.pulmon. immunization, whereas un-adjuvanted H56 rapidly drains to the local lymph nodes

**Figure 2:**
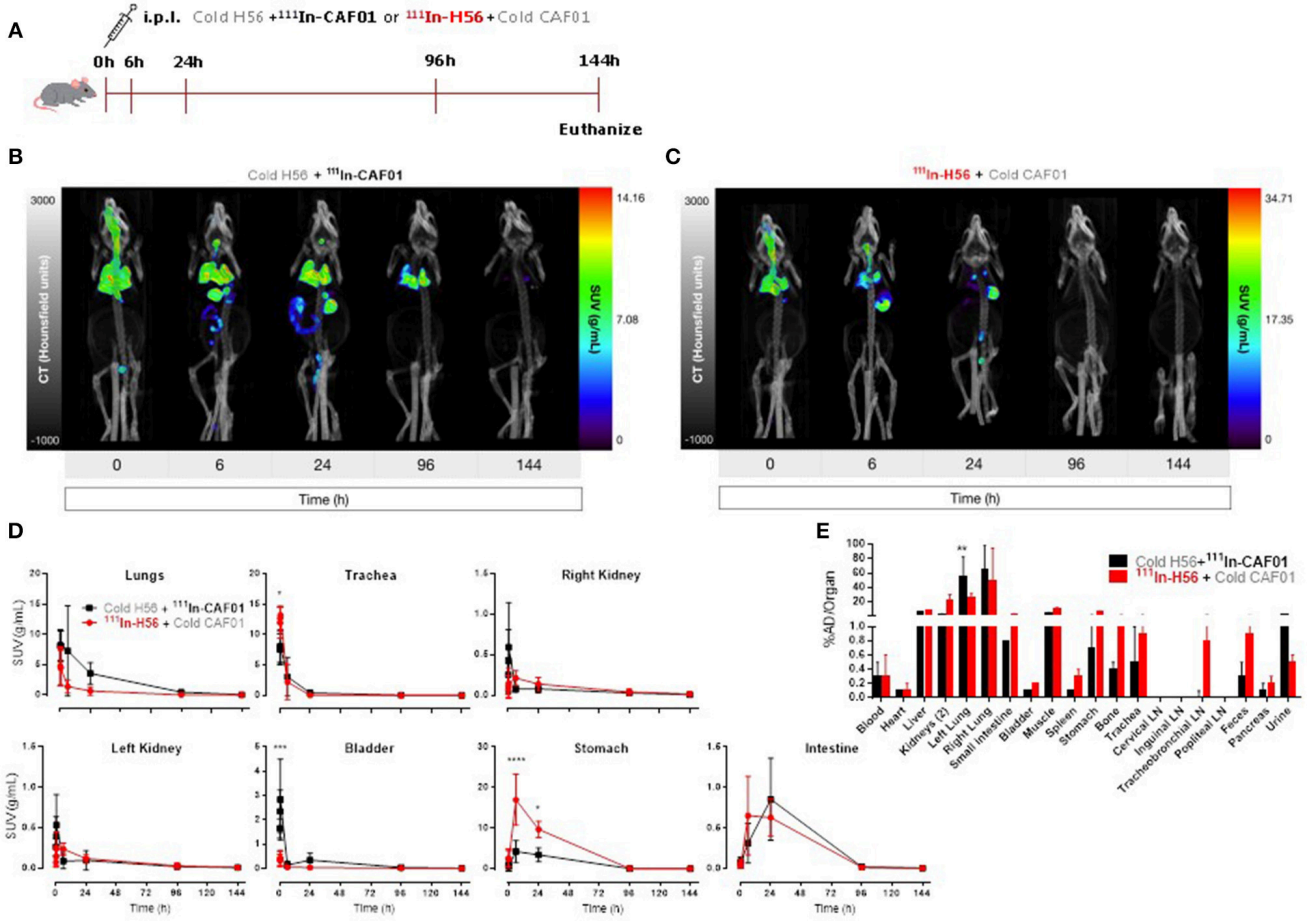
The H56/CAF01 vaccine remains in the lungs following i.pulmon. administration, whereas H56 drains to the local lymph nodes. **(A)** Experimental scheme: Mice were exposed i.pulmon. to 10 μg cold H56 adjuvanted with ^111^In-CAF01 (125/25 μg DDA/TDB) and 10 μg ^111^In-H56 adjuvanted with cold CAF01 (125/25 μg DDA/TDB), respectively. Animals were imaged by dynamic whole-body SPECT/CT scan for the initial 40 min (10 min/frame) and after that static 40 min scans at 6 and 24 h, 60 min scan at 96 h and 90 min scan at 144 h were conducted. Animals were euthanized on day 6 after immunization for *ex vivo* quantification of the biodistribution using a gamma counter. Representative SPECT/CT images of a mouse dosed i.pulmon. with cold H56 + ^111^In-CAF01 **(B)** or ^111^In-H56 + cold CAF01 **(C)** and imaged over 144 h post-immunization. **(D)** Organ SUVs in g/mL of the cold H56 + ^111^In-CAF01 or ^111^In-H56 + cold CAF01 over 144 h post-immunization; calculated from dynamic and static SPECT/CT images. Statistical analysis: two-way ANOVA and Sidak's post-test. Data represent mean values ± s.d., *n* = 3. *p < 0.05, ***p < 0.001, and ****p < 0.0001. **(E)** *Ex vivo* organ biodistribution [% administered dose (AD)/organ] of the cold H56 + ^111^In-CAF01 or ^111^In-H56 + cold CAF01 on day 6 (144 h) post-immunization. Statistical analysis: two-way ANOVA and Sidak's post-test. Bars represent mean values ± s.d., *n* = 3. **p < 0.01.

First, we evaluated the biodistribution and kinetics of the H56/CAF01 vaccine upon pulmonary administration. Mice were dosed i.pulmon. with cold H56 + ^111^In-CAF01 or ^111^In-H56 + cold CAF01, respectively, and the vaccine biodistribution and pharmacokinetics were visualized and quantified by SPECT/CT imaging at designated time points post-injection (Figure 2A). On day 6, the mice were euthanized, and the remaining activity was measured in different organs using a gamma counter. The SPECT/CT images permitted precise anatomical localization of ^111^In-CAF01 and ^111^In-H56 in the animals (Figures 2B,C). The images clearly reflect pronounced differences in the pharmacokinetics of H56 and CAF01 following i.pulmon. administration, and H56 was cleared much faster from the lungs than CAF01. Initially, the vaccine (H56 and CAF01) was apparently distributed evenly in the lungs, and it remained in the lungs during the first 30 min post-injection. Administration *via* the i.pulmon. route often results in deposition of a certain dose fraction of the radiopharmaceutical in the back of the mouth, which is subsequently swallowed (47). Hence, this dose fraction will redistribute to the stomach and the upper gastrointestinal tract, as observed in the 6 h scan. By 6 h, H56 as well as CAF01 were slowly cleared from the stomach and the intestines, and continued to transit in the gut up to 24 h after dosing. The H56 protein could only be detected in the animals up to 24 h post administration (Figure 2C), as compared to CAF01, which remained longer in the lungs and was detectable until termination of the experiment (day 6), although with a time-dependent decline in signal intensity (Figure 2B). Quantification of the *in vivo* biodistribution (SUV values) supported these observations (Figure 2D). The activity observed in the lungs for CAF01 was relatively higher than the activity measured for H56 during the entire experiment (Figure 2D, upper left). Both H56 and CAF01 showed very low activity in the trachea, kidneys and bladder at the designated time-points, except at the initial 30 min, where a high activity was detected. Notably, a higher activity was also measured in the stomach 6 and 24 h post injection for H56 than for CAF01, although there was no difference in the intestinal activity at these time points (Figure 2D, lower right). Whole body activity was measured on day 6 after euthanizing the animals and compared to the total administered dose, and the percentage of the administered dose per organ/tissue was calculated (Figure 2E). The SPECT/CT image analysis corroborate with the *ex vivo* biodistribution data, which showed that the major part of the radioactive dose was recovered in the lungs (Figure 2E). However, there was a statistically significant difference between the remaining activity of H56 and CAF01 in the left lung. For CAF01, a relatively low dose fraction of the radioactivity was found in the liver, kidneys and muscle. In contrast, H56 was detectable in relatively higher amounts in the liver and the kidneys, confirming a faster metabolism and elimination of H56 than CAF01. A comparatively higher activity of H56 than CAF01 was also observed in other organs, including the lung-draining LNs, which confirms the pronounced differences in the biodistribution and pharmacokinetics of H56 and CAF01.

### The vaccine forms a depot following parenteral (s.c.) administration

**Figure 3:**
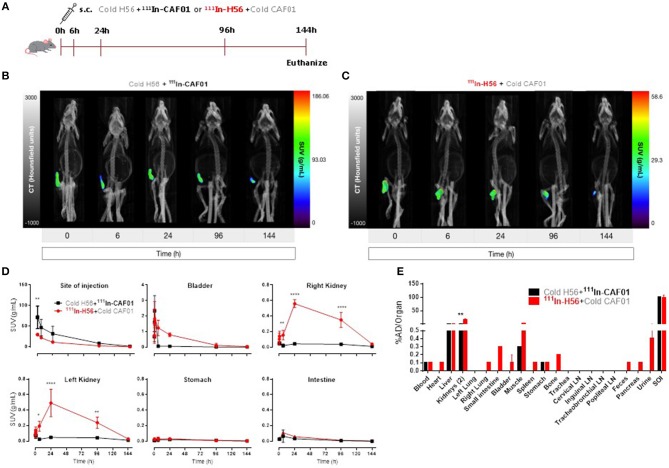
The H56/CAF01 vaccine forms a depot at the site of injection following s.c. administration. **(A)** Experimental scheme: S.C. immunization was carried out with 10 μg cold H56 adjuvanted with ^111^In-CAF01 (125/25 μg DDA/TDB) or 10 μg ^111^In-H56 adjuvanted with cold CAF01 (125/25 μg DDA/TDB). Dynamic whole-body SPECT/CT scans were carried out for 40 min (10 min/frame) and thereafter static 40 min scans at 6 and 24 h, 60 min scan at 96 h and 90 min scan at 144 h were performed. Animals were euthanized on day 6 after immunization for *ex vivo* quantification of the biodistribution using a gamma counter. Representative SPECT/CT images of a mouse injected s.c. with cold H56 + ^111^In-CAF01 **(B)** and ^111^In-H56 + cold CAF01 **(C)**, imaged over 144 h post-administration. **(D)** Organ SUVs in g/mL of the cold H56 + ^111^In-CAF01 or ^111^In-H56 + cold CAF01 over 144 h post-immunization; calculated from dynamic and static SPECT/CT images. Statistical analysis: two-way ANOVA and Sidak's post-test. Data represent mean values ± s.d., *n* = 3. *p < 0.05, **p < 0.01, and ****p < 0.0001. **(E)** *Ex vivo* organ biodistribution (% administered dose (AD)/organ] of the cold H56 + ^111^In-CAF01 or ^111^In-H56 + cold CAF01 on day 6 (144 h) post-immunization. Statistical analysis: two-way ANOVA and Sidak's post-test. Bars represent mean values ± s.d., *n* = 3. **p < 0.01.

For control and comparative purposes, we examined the biodistribution of the H56/CAF01 vaccine following parenteral administration. Mice were injected s.c. with either ^111^In-CAF01 or ^111^In-H56 with cold H56 or cold CAF01, respectively, and the vaccine biodistribution was visualized and quantified by SPECT/CT imaging at the designated time-points up to 144 h post-immunization (Figure 3A). Mice were euthanized 144 h post-injection for *ex vivo* quantification of the biodistribution. The SPECT/CT images clearly showed ^111^In-CAF01 and ^111^In-H56, respectively, in the animals at the site of injection (Figures 3B,C). CAF01 and H56 displayed a highly comparable biodistribution after s.c. administration, and the major part of the dose remained at the injection site for the entire duration of the experiment, suggesting the formation of a vaccine depot at the site of injection, as previously published (48). However, the activity of ^111^In declined during the experiment to a minimal level 144 h post-injection. A high activity was observed at the injection site for both CAF01 and H56 (Figure 3D), which declined until 144 h (Figure 3D, upper left). However, a significantly lower activity was detected for H56, as compared to CAF01, up to 30 min post-injection. Due to their relatively large size and cationic charge, CAF01 liposomes stayed at the injection site as previously published (48), and almost no activity of ^111^In-CAF01 was observed in the kidneys and the bladder post-injection. In contrast, H56 was cleared faster from the injection site and was subsequently excreted through the kidneys, which was evident from the 6, 24, and 96 h post-injection images (Figure 3D, upper right and lower left) and a correspondingly higher activity for H56 in the bladder than CAF01 (Figure 2D, upper middle). The initial sharp increase in ^111^In activity within 30 min post-injection was due to free ^111^In (Figure 2D, upper middle) in the radiopharmaceutical. Almost no activity was observed in the stomach and the intestines following s.c. immunization (Figure 3D, lower middle and right). As expected, most of the radioactivity was detected at the site of injection (Figure 3E). For CAF01, a relatively low dose fraction of radioactivity was found in the liver, kidneys, and muscle, which was collected from the right thigh close to the site of injection. In contrast, radioactivity for H56 was observed in several other organs besides the liver, kidney and muscle. Comparing the two, the radioactivity in the kidneys was statistically significantly higher for H56 than for CAF01, which correlates well with the SPECT/CT image analyses. Hence, the SPECT/CT and the *ex vivo* biodistribution data collectively confirm that the major dose fraction of CAF01 stays at the site of injection following s.c. injection, while a small dose fraction of H56 is cleared slowly from the injection site into the blood stream and is subsequently excreted through the kidneys.

### Combined parenteral (i.m.) prime and airway (i.pulmon.) boost immunizations increase H56-specific IgA titers in the airways

**Figure 4:**
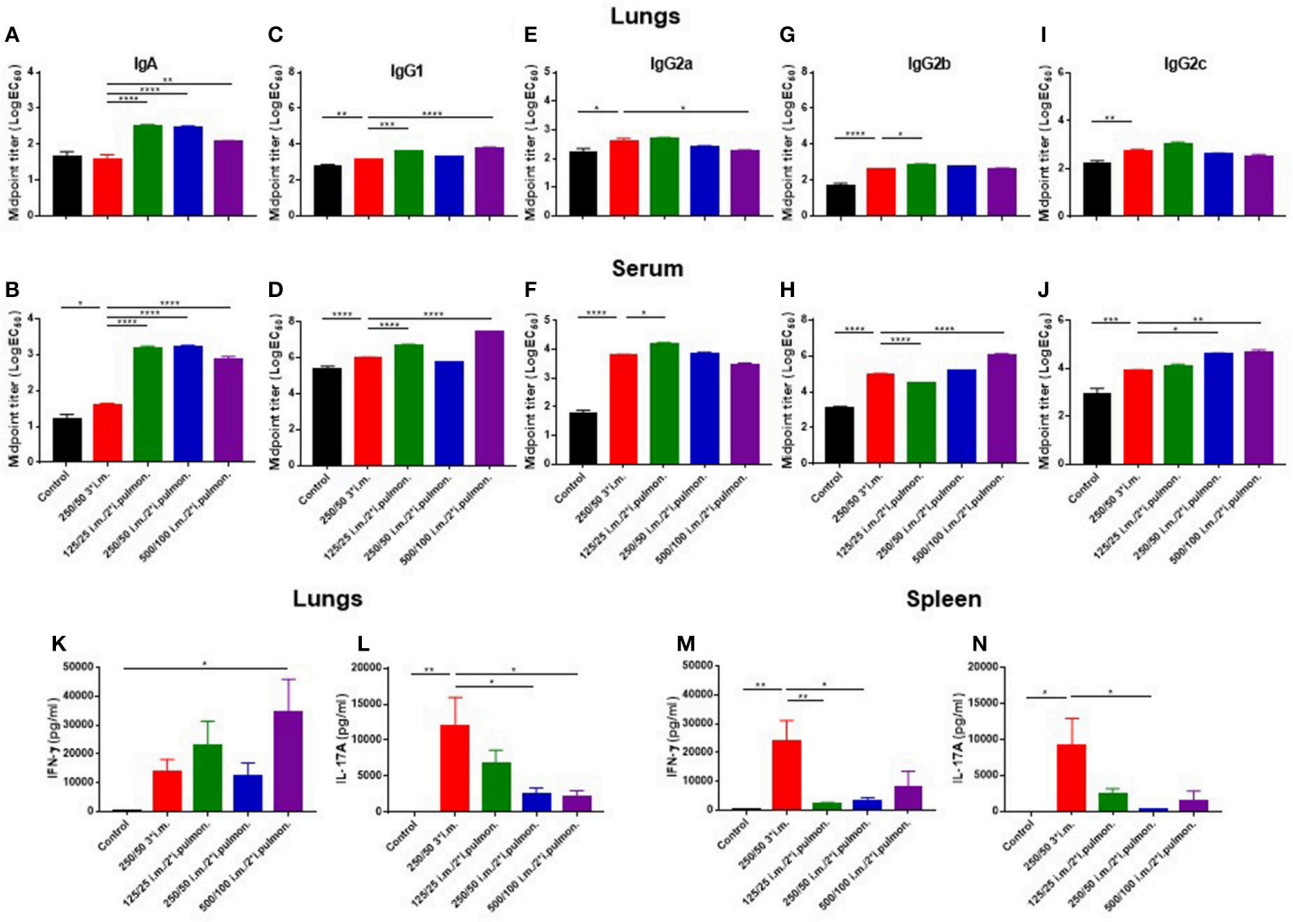
Strong antibody and cytokine responses in the lungs and serum after parenteral (i.m.) prime and airway (intrapulmonary, i.pulmon.) mucosal boost immunization with H56/CAF01. Mice were primed i.m. and boosted twice i.m. (5/250/50 μg H56/DDA/TDB) or primed i.m. (5/250/50 μg H56/DDA/TDB) and boosted twice i.pulmon. (10/125/25 or 10/250/50 or 10/500/100 μg H56/DDA/TDB) at 2 weeks interval with H56 adjuvanted with different doses of CAF01 (DDA/TDB). IgA **(A,B)**, IgG1 **(C,D)**, IgG2a **(E,F)**, IgG2b **(G,H)**, and IgG2c **(I,J)** mid-point titers (log EC_50_ values) were determined in homogenized lung supernatants and serum at 2 weeks after the last boost immunization. Lung cells **(K,L)** and splenocytes **(M,N)** were isolated 2 weeks after the last boost immunization and *in vitro* stimulated with H56 for 72 h and IFN-γ and IL-17 levels were determined by ELISA. Statistical analysis: one-way ANOVA and Tukey's post-test. Bars represent mean values ± s.e.m., *n* = 6. *p < 0.05, **p < 0.01, ***p < 0.001, and ****p < 0.0001.

With the objective to establish an immunization protocol that generates strong local IgA responses in the airways, we compared vaccine-induced lung IgA responses using two different vaccination strategies; i.m. prime/i.m. boost and i.m. prime/i.pulmon. boost immunization. Mice were immunized twice with CAF01-adjuvanted H56 *via* the i.m./i.m. or i.m./i.pulmon. administration routes, respectively, using different doses of CAF01 to determine the optimal adjuvant dose. Subsequently, we measured H56-specific IgA, IgG1, IgG2a, IgG2b, IgG2c, and IgM antibodies in the lung homogenate supernatants, as well as the IFN-γ and IL-17 production by H56-restimulated lung cells 2 weeks after the last booster immunization. For the antibody responses, the mid-point titers were calculated from O.D. values measured by ELISA. Significantly higher levels of H56-specific IgA responses in the lungs (Figure 4A) and the serum (Figure 4B) were measured for the three groups immunized i.m./i.pulmon, as compared to the levels measured for mice immunized three times i.m. Similarly, the lung (Figure 4C) and serum IgG1 responses (Figure 4D) were significantly higher following i.m./i.pulmon. immunization using the lowest (125/50 μg DDA/TDB) and the highest CAF01 dose (500/100 μg DDA/TDB) than 250/50 3^*^i.m. immunization. For the other IgG isotype responses in the lungs (Figures 4E,G,I), no difference was observed between i.m./i.m. vs. i.m./i.pulmon. immunization schedules, except for the IgG2b responses (Figure 4G), which were higher for the animals immunized using the i.m./i.pulmon. than for the animals immunized using i.m./i.m. schedule. However, the lung IgG2a responses were lower for 500/100 i.m./2^*^i.pulmon. as compared to 250/50 3^*^i.m. immunization (Figure 4E). In general, the serum H56-specific IgG isotype production was higher for all three groups immunized i.m./ i.pulmon., as compared to the titers for the group immunized three times i.m. (Figures 4D,**F,H,J**). However, the H56-specific IgG2b responses were lower in the 125/25 i.m./2^*^sngsjehdlejhdx, ki.pulmon. group (Figure 4H). As expected, IgM was only detected at low levels in serum and lung homogenates (Supplementary Figure S1).

Correlating with these findings, i.m./i.pulmon. immunization induced high IFN-γ levels in the lungs (Figure 4K), and the IFN-γ levels were significantly higher for mice vaccinated once i.m. followed by 500/100 i.m./2^*^i.pulmon. as compared to three i.m. immunizations (250/50). On the other hand, the IL-17 responses in the lungs were significantly lower for both 250/50 i.m./2^*^i.pulmon. and 500/100 i.m./2^*^i.pulmon. than 250/50 3^*^i.m. immunization (Figure 4L). However, the 125/25 i.m./2^*^i.pulmon. immunization-induced IL-17 responses were not significantly different from the responses for mice immunized three times i.m. In contrast to the local lung responses, the systemic IFN-γ and IL-17 responses measured in serum were significantly higher for the mice immunized three times i.m. (250/50) as compared to all three i.m./i.pulmon. immunizations (Figures 4M,N). Overall, i.m./i.pulmon. immunization induced higher local and systemic antibody and dose-dependent equivalent cytokine responses than 250/50 3^*^i.m. immunization.

### Parenteral (i.m.) prime and airway (i.pulmon.) boost immunization cause localization of H56-specific Th1 and Th17 cells in the lung parenchyma

**Figure 5:**
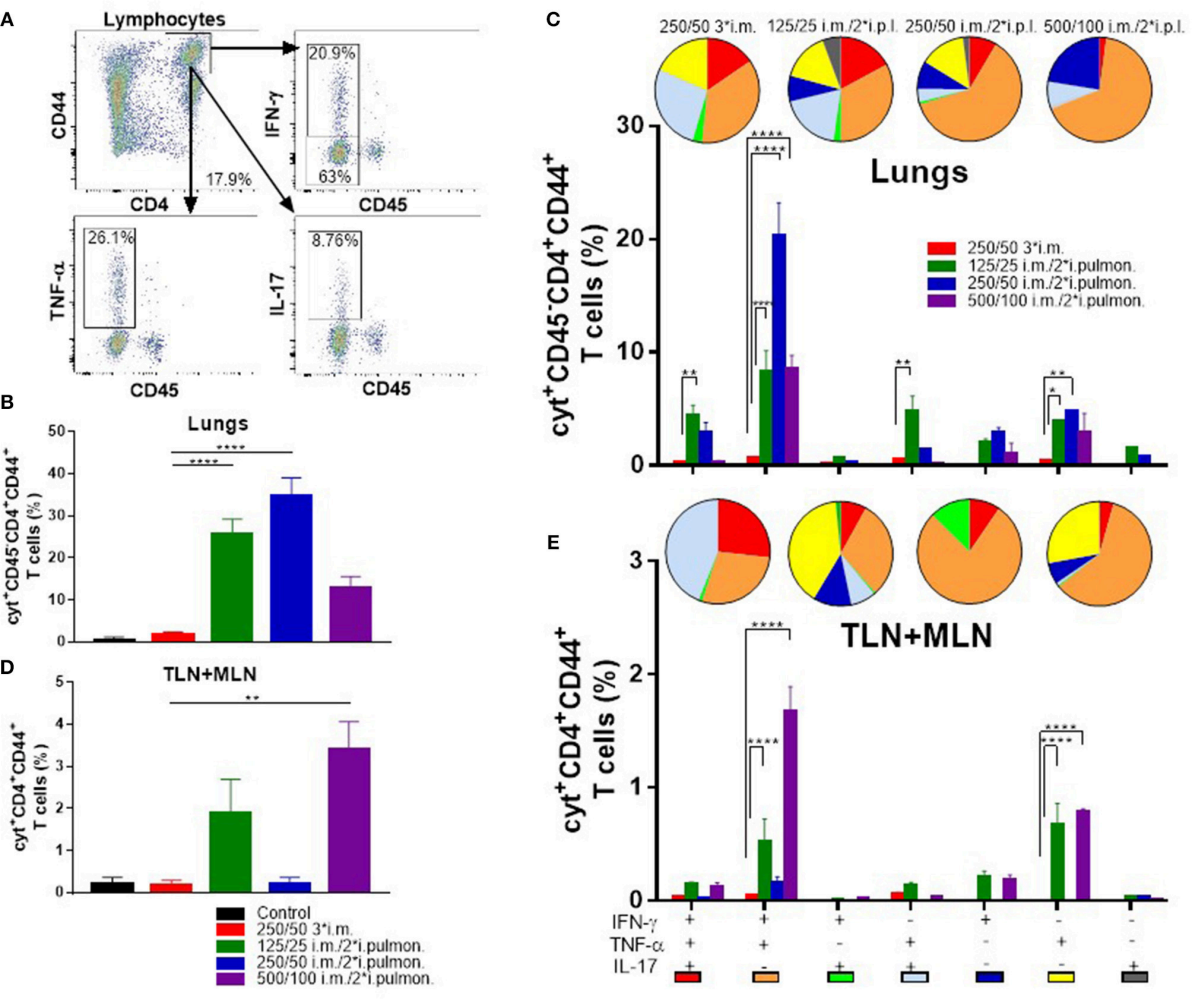
Robust T-cell responses in the lungs and the lung-draining tracheobronchial and mediastinal lymph nodes (TLN + MLN) by parenteral (i.m.) priming and airway (intrapulmonary, i.pulmon.) mucosal H56/CAF01 immunization. Mice were primed i.m. and boosted twice i.m. (5/250/50 μg H56/DDA/TDB) or primed i.m. (5/250/50 μg H56/DDA/TDB) and boosted twice i.pulmon. (10/125/25 or 10/250/50 or 10/500/100 μg H56/DDA/TDB) at 2 weeks interval with H56 adjuvanted with different concentrations of CAF01 (DDA/TDB). Lung cells and the lung draining LNs (TLN + MLN) were examined for CD4^+^CD44^+^ T cells and IFN-γ, TNF-α, and IL-17 cytokines by intracellular flow cytometry analysis after stimulation with H56, 2 weeks after the last booster immunization. **(A)** Gating strategy for quantification and localization of lung-associated CD44^+^ T cells in immunized mice (exemplified by lung cells from an immunized mouse from the vaccine group 125/25 i.m./2*i.pulmon.). Lung cells were examined for labeling with i.v. injected FITC-conjugated anti-CD45.2 mAb 2 weeks after the last booster immunization by intracellular flow cytometry analysis after stimulation with H56. Dot plot shows IFN-γ, TNF-α, or IL-17 expression in i.v.CD45^+^ (intravascular) and i.v.CD45^−^ (parenchymal) cells. **(B)** Number of cyt^+^CD4^+^CD44^+^ T cells (any cytokine IFN-γ, TNF-α, and IL-17), which are located in lung parenchyma (CD45^−^). Statistical analysis: one-way ANOVA and Tukey's post-test. Bars represent mean values ± s.e.m., *n* = 6. ****p < 0.0001. **(C)** After Boolean gating analysis, the frequencies of the seven possible CD4^+^CD44^+^ T cell subpopulations expressing any combination of the IFN-γ, TNF-α, and IL-17 cytokines are shown for all immunization groups. The background from the control group was subtracted. Pie charts represent the fraction of CD4^+^CD44^+^ T cells expressing different cytokine combinations. Pie chart color-coding and the subpopulation association for each color is shown below the bar graph **(E)**. Statistical analysis: two-way ANOVA and Tukey's post-test. Bars represent mean values ± s.e.m., *n* = 6. *p < 0.05, **p < 0.01, and ****p < 0.0001. **(D)** Number of cyt^+^CD4^+^CD44^+^ T cells (any cytokine IFN-γ, TNF-α, and IL-17) in the lung draining LNs (TLN+MLN). Statistical analysis: one-way ANOVA and Tukey's post-test. Bars represent mean values ± s.e.m., *n* = 6. **p < 0.01. **(E)** Boolean gating analysis and pie charts of CD4^+^CD44^+^ T cells expressing different cytokine combinations in the lung-draining LNs (TLN+MLN), 2 weeks after the last booster immunization. Statistical analysis: two-way ANOVA and Tukey's post-test. Bars represent mean values ± s.e.m., *n* = 6. ****p < 0.0001.

Subsequently, we measured in further detail the T-cell recruitment, which takes place in i.m.-primed animals during airway mucosal boost (i.pulmon.) immunization, as compared to animals receiving an i.m. boost (Figure 5A). First, we examined, if H56-specific T cells were localized in the lung parenchyma or in the lung vasculature by subjecting immunized mice to *in vivo* intravascular staining. A FITC-labeled anti-CD45 mAb was injected i.v. 3 min before euthanizing the animals, which resulted in FITC staining of all intravascular, but not parenchymal lymphocytes, as previously described (49). Subsequently, we identified the i.v. CD45^−^ and CD45^+^ populations of H56-specific CD4^+^CD44^+^ T cells producing IFN-γ, TNF-α and IL-17, respectively, 2 weeks after the last immunization by intracellular cytokine staining. The i.m./i.pulmon. immunization strategy caused markedly elevated infiltration of CD4^+^CD44^+^ T cells in the lungs, as compared to i.m./i.m. immunization (Figures 5B,C). Most of these cells were localized in the lung parenchyma, and the percentage of IFN-γ, TNF-α, or IL-17 cytokine-producing i.v.CD45^−^ cells was significantly higher for 125/25 i.m./2^*^i.pulmon. and 250/50 i.m./2^*^i.pulmon. than 250/50 3^*^i.m. group (Figure 5B). A similar trend was observed when we examined the frequencies of each of the single cytokine-producing i.v. CD45^−^CD4^+^CD44^+^ T cells among the different immunization groups (Supplementary Figure S2A). The functionality of the antigen-specific CD4^+^CD44^+^ T cells was determined with respect to their expression of IFN-γ, TNF-α and IL-17, respectively, or their combinations, for both immunization strategies by combinatorial Boolean gating analysis (Figure 5C). The polyfunctionality of the CD4^+^CD44^+^ T cells is represented pictorially by pie charts after deduction of control group responses from all immunization groups. The i.m./i.pulmon. immunization induced primarily polyfunctional T-cell populations consisting of double (IFN-γ^+^TNF-α^+^ and TNF-α^+^IL-17^+^) and triple (IFN-γ^+^TNF-α^+^IL-17^+^) positive cytokine-producing memory CD4^+^CD44^+^ T cells and a lower frequency of single (IFN-γ^+^, TNF-α^+^, or IL-17^+^) cytokine-positive effector CD4^+^CD44^+^ T cells (Figure 5C, pies). This shows that there is a CAF01 dose-dependent increase in the frequency of terminally differentiated effector CD4^+^CD44^+^ T cells producing IFN-γ^+^ alone (Figure 5C, blue pies), whereas immunization with a lower dose of CAF01 led to more IL-17 producing memory-line T cells also expressing TNF-α with or without expression of IFN-γ (Figure 5C, red and light blue pies). However, there was a dose-dependent increase in the IFN-γ^+^TNF-α^+^ producing CD4^+^CD44^+^ T cells (Figure 5C, orange pies). In agreement with the stronger antibody and cytokine responses measured above, there was a higher frequency of H56-specific, cytokine-producing memory CD4^+^CD44^+^ T cells in the lungs following i.m./i.pulmon. immunization, as compared to the 250/50 3^*^i.m. immunization, which induced very low levels of these cells (Figure 5C, bars). Among the three groups immunized using i.m./i.pulmon. vaccination, immunization at the lowest CAF01 dose (125/25 μg DDA/TDB) induced consistently higher frequencies of cytokine-producing single, double, and triple cytokine-producing CD4^+^CD44^+^ T cells, except IFN-γ^+^TNF-α^+^ subgroup, where immunization with 250/50 μg DDA/TDB induced the highest frequency of IFN-γ^+^TNF-α^+^ producing CD4^+^CD44^+^ T cells.

T cells were also measured in the lymph nodes draining the lungs (TLNs + MLNs) in immunized mice (Figures 5D,E). I.m./2^*^i.pulmon. immunization with 125/25 and 500/100 μg DDA/TDB led to a markedly higher frequency of IFN-γ, TNF-α, and IL-17 cytokine-producing CD4^+^CD44^+^ T cells as compared with 250/50 3^*^i.m. immunization (Figure 5D). At the individual cytokine level, i.m./i.pulmon. immunization with the highest CAF01 dose (500/100 μg DDA/TDB) induced the highest CD4^+^CD44^+^ T-cell levels (Supplementary Figure S2B). The i.m./i.pulmon. immunization induced a polyfunctional T-cell population consisting of single (IFN-γ^+^ and TNF-α^+^, respectively) cytokine-positive effector CD4^+^CD44^+^ T cells, double (IFN-γ^+^TNF-α^+^ and TNF-α^+^IL-17^+^) positive, cytokine-producing memory CD4^+^CD44^+^ T cells and triple (IFN-γ^+^TNF-α^+^IL-17^+^) cytokine-positive CD4^+^CD44^+^ T cells (Figure 5E, pies). For the groups immunized i.m./i.pulmon., there was a dose-dependent decrease in the relative frequency of double-positive (TNF-α^+^IL-17^+^) and triple-positive CD4^+^CD44^+^ T cells (Figure 5E, red pies). In contrast to observations in the lungs, the IFN-γ^+^TNF-α^+^ producing CD4^+^CD44^+^ T cells in the draining lymph nodes did not show a dose-dependent increase after i.m./i.pulmon. immunization, and the highest fraction of cells was observed after i.m./i.pulmon. immunization with 250/50 μg DDA/TDB (Figure 5E, orange pies). As in the lungs, noticeably higher frequencies of memory CD4^+^CD44^+^ T cells were observed in the TLNs and MLNs following i.m./i.pulmon. immunization, as compared to the frequencies after i.m. immunization (Figure 5E, bars), and immunization with 125/25 μg DDA/TDB induced higher frequencies of all subsets of cytokine-producing CD4^+^CD44^+^ T cells, except for the IFN-γ^+^TNF-α^+^ producing subset.

### H56-specific Th1 and Th17 cells are induced in the spleen upon parenteral (i.m.) prime and airway (i.pulmon.) boost immunization

**Figure 6:**
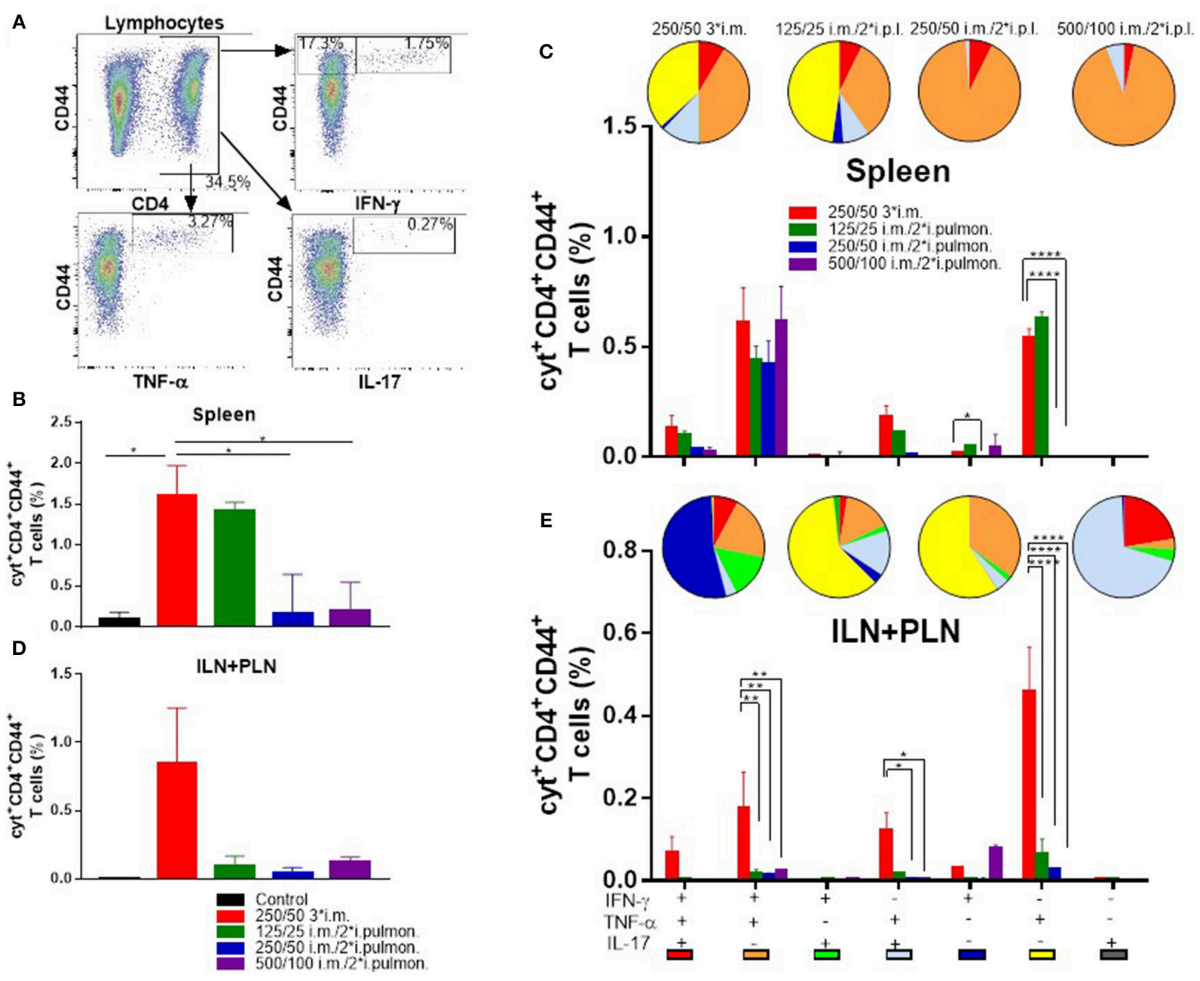
Parenteral (i.m.) prime and airway (i.pulmon.) mucosal boost immunization with H56/CAF01 induce equivalent T-cell responses in the spleen as parenteral (i.m.) prime-boost immunization. Mice were primed i.m. and boosted twice i.m. (5/250/50 μg H56/DDA/TDB) or primed i.m. (5/250/50 μg H56/DDA/TDB) and boosted twice i.pulmon. (10/125/25 or 10/250/50 or 10/500/100 μg H56/DDA/TDB) at 2 weeks interval with H56 adjuvanted with different concentrations of CAF01 (DDA/TDB). Spleen cells and the inguinal and popliteal lymph nodes (ILN+PLN) draining the site of i.m. injection were harvested 2 weeks after the last booster immunization, surface-stained for the CD4 and CD44 receptors and intracellular localized IFN-γ, TNF-α, and IL-17 cytokines by intracellular flow cytometry analysis after stimulation with H56. **(A)** Gating strategy for the evaluation of antigen-specific, cytokine-producing CD44^+^ T cells in spleen and lymph nodes of immunized mice (exemplified by TLN + MLN cells from an immunized mouse from the vaccine group 125/25 i.m./2*i.pulmon.). Dot plot shows IFN-γ, TNF-α, or IL-17 expression in CD44^+^ T cells. **(B)** Number of cyt^+^CD4^+^CD44^+^ T cells (any cytokine IFN-γ, TNF-α, and IL-17) in the spleen. Statistical analysis: one-way ANOVA and Tukey's post-test. Bars represent mean values ± s.e.m., *n* = 6. *p < 0.05. **(C)** After a Boolean gating analysis, the frequencies of the seven possible CD4^+^CD44^+^ T cell subpopulations expressing any combination of the IFN-γ, TNF-α, and IL-17 cytokines are shown for all immunization groups. Background from the control group was subtracted. Pie charts represent the fraction of CD4^+^CD44^+^ T cells expressing different cytokine combinations. Pie chart color-coding and the subpopulation association for each color is shown below the bar graph **(E)**. Statistical analysis: two-way ANOVA and Tukey's post-test. Bars represent mean values ± s.e.m., *n* = 6. *p < 0.05 and ****p < 0.0001. **(D)** Number of cyt^+^CD4^+^CD44^+^ T cells (any cytokine IFN-γ, TNF-α and IL-17) in LNs draining the site of injection (ILN + PLN). Bars represent mean values ± s.e.m., *n* = 6. **(E)** Boolean gating analysis and pie charts of CD4^+^CD44^+^ T cells expressing different cytokine combinations in the LNs draining the site of injection (ILN+PLN) 2 weeks after the last booster immunization. Statistical analysis: two-way ANOVA and Tukey's post-test. Bars represent mean values ± s.e.m., *n* = 6. *p < 0.05, **p < 0.01 and ****p < 0.0001.

Systemic induction of H56-specific Th1 and Th17 cells in the spleen was also investigated (Figure 6A). In general, the i.m./i.pulmon. vaccination strategy resulted in induction of lower relative frequencies of CD4^+^CD44^+^ T cells in the spleen, as compared to i.m./i.m. immunization (Figures 6B,**C**). Interestingly, immunization with 125/25 μg DDA/TDB generated equivalent frequencies of cytokine-producing T cells as the 250/50 3^*^i.m. immunization (Figure 6B). When comparing the frequencies of CD4^+^CD44^+^ T cells producing IFN-γ, TNF-α, and IL-17, respectively, we observed that i.m./i.pulmon. immunization with 125/25 and 250/50 μg DDA/TDB stimulated equivalent frequencies of T cells as the i.m. immunization with 250/50 μg DDA/TDB (Supplementary Figure S2C). Examination of the functionality of the H56-specific CD4^+^CD44^+^ T in the spleen revealed that i.m./i.pulmon. immunization with the lowest CAF01 dose (125/25 μg DDA/TDB) induced primarily a polyfunctional T cell population consisting of double (IFN-γ^+^TNF-α^+^ and TNF-α^+^IL-17^+^) and triple (IFN-γ^+^TNF-α^+^IL-17^+^) positive, cytokine-producing memory CD4^+^CD44^+^ T cells and TNF-α^+^ memory CD4^+^CD44^+^ T cells (Figure 6C, pies). Three i.m. immunizations promoted a comparable functionality profile. On the other hand, higher doses of CAF01 administered i.m./i.pulmon. (250/50 and 500/100 μg DDA/TDB, respectively) led to a dominant IFN-γ^+^TNF-α^+^ producing CD4^+^CD44^+^ T cell population (Figure 6C, orange pies). Unlike for the lungs, i.m./i.pulmon. immunization induced comparable frequencies of H56-specific, cytokine-producing memory CD4^+^CD44^+^ T cells in the spleen as the i.m./i.m. immunization (Figure 6C, bars). i.m. and i.m./2^*^i.pulmon. immunization with 125/25 μg DDA/TDB also promoted similar frequencies of IFN-γ^+^ or TNF-α^+^ producing effector CD4^+^CD44^+^ T cells.

Vaccine-induced T cells were also evaluated in the ILNs and PLNs draining the site of i.m. injection (Figure 6A), and we compared i.m./i.pulmon. vs. i.m./i.m. immunization, respectively. The i.m./i.m. immunization with 250/50 μg DDA/TDB induced higher but statistically indifferent cytokine-producing frequencies of CD4^+^CD44^+^ T cells in the draining LNs, as compared with i.m./i.pulmon. immunization (Figure 6D). There was significant differences in the frequencies of CD4^+^CD44^+^ T cells producing IFN-γ, TNF-α, or IL-17 between the groups vaccinated by i.m./i.pulmon., as compared to i.m./i.m. immunization with 250/50 μg DDA/TDB (Supplementary Figure S2D). However, there was a considerable difference in the functionality of the CD4^+^CD44^+^ T cells between the two immunization strategies (Figure 6E, pies). Whereas i.m./i.pulmon. immunization predominantly induced single cytokine-producing CD4^+^CD44^+^ T cells, i.m. immunization with 250/50 μg DDA/TDB resulted in a polyfunctional T-cell population consisting of double (IFN-γ^+^TNF-α^+^ and TNF-α^+^IL-17^+^) and triple (IFN-γ^+^TNF-α^+^IL-17^+^) positive, cytokine-producing memory CD4^+^CD44^+^ T cells and IFN-γ^+^ or TNF-α^+^ effector CD4^+^CD44^+^ T cells. I.m./i.pulmon. immunization did mainly induce very low frequencies of memory CD4^+^CD44^+^ T cells and promoted single cytokine-producing effector CD4^+^CD44^+^ T cells (Figure 6E, bars). The frequencies of memory and effector CD4^+^CD44^+^ T cells were significantly higher after i.m. immunization with 250/50 μg DDA/TDB, as compared to the frequencies detected after i.m./i.pulmon. immunization.

### The biodistribution of parenteral (s.c.) prime and airway (i.pulmon.) boost administered vaccine mimics the biodistribution of i.pulmon. administered vaccine

**Figure 7:**
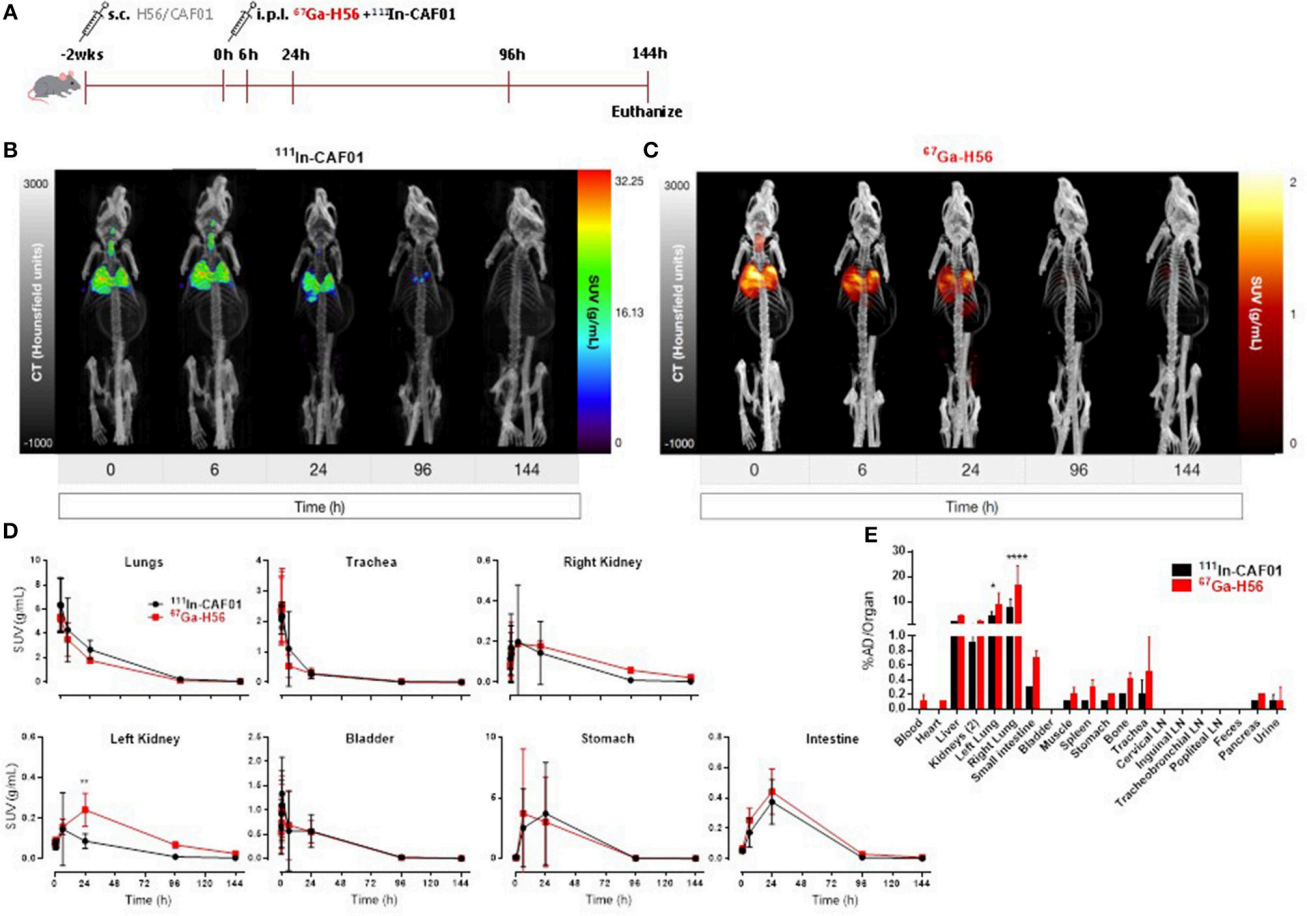
H56/CAF01 vaccine biodistribution upon parenteral (s.c.) priming and airway (i.pulmon.) mucosal boosting follow a similar trend as the biodistribution following airway (i.pulmon.) mucosal prime immunization. **(A)** Experimental scheme: Mice were prime-immunized s.c. with 5 μg cold H56 adjuvanted with cold CAF01 (250/50 μg DDA/TDB). At week 2, animals were boost-immunized *via* intrapulmonary (i.pulmon.) route with 10 μg ^67^Ga-H56 adjuvanted with ^111^In-CAF01 (125/25 μg DDA/TDB). Animals were imaged by dynamic whole-body SPECT/CT scan for the initial 40 min (10 min/frame) and after that static 40 min scans at 6 and 24 h, 60 min scan at 96 h and 90 min scan at 144 h were conducted. Animals were euthanized on day 6 after immunization for *ex vivo* biodistribution using a gamma counter. Representative SPECT/CT images showing biodistribution of ^111^In-CAF01 **(B)** and ^67^Ga-H56 **(C)** of a mouse prime-immunized s.c. with H56/CAF01 and boost-immunized i.pulmon. with ^67^Ga-H56 + ^111^In-CAF01. **(D)** Organ SUVs (g/mL) were calculated from dynamic and static SPECT/CT images of the cold s.c. prime-immunized and hot (^67^Ga-H56 + ^111^In-CAF01) i.pulmon. boost-immunized animals over 144 h post-immunization. Statistical analysis: two-way ANOVA and Sidak's post-test. Data represent mean values ± s.d., *n* = 3. **p < 0.01. **(E)** *Ex vivo* organ biodistribution [% administered dose (AD)/organ] of the cold s.c. prime-immunized and hot (^67^Ga-H56 + ^111^In-CAF01) i.pulmon. boost-immunized animals on day 6 (144 h) post-immunization. Statistical analysis: two-way ANOVA and Sidak's post-test. Bars represent mean values ± s.d., *n* = 3. *p < 0.05 and ****p < 0.0001.

We used SPECT/CT to investigate the biodistribution of the H56/CAF01 vaccine following parenteral prime-airway mucosal boost immunization and compared with our previous biodistribution results. We performed this study as we wanted to know whether there would be a faster clearance of H56 or CAF01 on i.pulmon. immunization with previously primed animals. Mice were primed s.c. with cold H56 or cold H56/CAF01, respectively, and boosted i.pulmon. 2 weeks later with ^67^Ga-H56 or ^67^Ga-H56/^111^In-CAF01, respectively. Mice were imaged by SPECT/CT imaging at the designated time-points up to 144 h post-injection, and the vaccine biodistribution was visualized and quantified (Figure 7A and Supplementary Figure S3A), followed by terminal e*x vivo* quantification of the biodistribution on day 6 of the study. Dual-isotope labeling of H56 and CAF01 with ^111^In and ^67^Ga, respectively, followed by SPECT-CT imaging, allowed for anatomical visualization of vaccine uptake in the lungs, as well as biodistribution and pharmacokinetics (Figures 7B,C). The images demonstrate pronounced differences in the biodistribution of H56 and CAF01 following s.c. prime and i.pulmon. boost immunization, where H56 was cleared within 24 h post-injection. The i.pulmon. administration of unadjuvanted H56 resulted in a very fast clearance of H56 within 6 h (Supplementary Figure S3B). The vaccine remained in the lungs for up to 6 h, followed by a slow redistribution to the stomach and intestines up to 24 h of the study. The signal from H56 was clearly visible until 24 h (Figure 7C) as compared to CAF01, which could be weakly visualized up to 96 h post-injection (Figure 7B). The *in vivo* quantification of radioactivity in images through SUV values verify these findings (Figure 7D). In contrast to a single i.pulmon. administration of the H56/CAF01 vaccine, s.c. prime-i.pulmon. boost immunization resulted in comparable activity of H56 and CAF01 in the lungs at the designated time points (Figure 7D, upper left). A relatively higher activity for both H56 and CAF01 was observed in the trachea, kidneys and bladder within 30 min post-injection, which declined at later time-points. As observed previously, a high activity of both H56 and CAF01 was observed in the stomach and the intestines at 6 and 24 h post-injection. The SUVs for unadjuvanted H56 showed a very low activity in the lungs and other organs, and most of the activity was observed in the kidneys within 6 h, which reflects that immunization with unadjuvanted H56 leads to lower retention in the lungs and faster metabolism and elimination of the protein (Supplementary Figure S3C). Whole-body activity was measured 144 h post-i.pulmon.-boost immunization. The results are in line with the imaging-based *in vivo* biodistribution, and the major fraction of the radioactivity was observed in the lungs (Figure 7E). The biodistribution profile of unadjuvanted H56 showed relatively low H56 activity in the lungs and liver (Supplementary Figure S3D). For H56/CAF01 s.c. prime- i.pulmon. boost immunization, there were differences in the remaining activity *ex vivo* between H56 and CAF01 in the lungs with comparatively higher radioactivity for H56 than for CAF01. For all other organs than the lungs, a relatively low dose fraction of CAF01-associated radioactivity was found in the liver, kidneys and intestine, as observed previously following either a single s.c. or i.pulmon. administration (Figure 7E). In contrast, H56 radioactivity was detectable in relatively higher dose fractions in the liver and kidneys, which supports our prior observations of a faster clearance of H56 than CAF01 (Figure 7E). Proportionately higher amounts of H56 activity was also observed in organs other than the liver and the kidneys. However, no remaining activity was detected in the lung-draining LNs, as observed earlier. We also compared the activity of ^67^Ga-H56 alone with ^67^Ga-H56 adsorbed to ^111^In-CAF01 and observed significant differences in the pulmonary uptake and biodistribution of unadjuvanted protein, as compared to liposome-adsorbed protein (Supplementary Figure S4). Unadjuvanted H56 was cleared much faster than the CAF01-bound H56.

## Discussion

Although a more efficacious vaccine is urgently needed for TB, vaccine design and development has been very challenging due to the specific requirement for induction of cell-mediated and mucosal immunity (2). It is now widely accepted that it is beneficial to stimulate Th1 and Th17 CD4^+^ T-cell responses in the lungs for vaccine-induced protection in general (50, 51) and against *Mtb* infection in particular (52, 53). *Mtb* evades host immunity by delaying the extravasation of primed circulating antigen-specific T cells into the lung mucosa (2, 54). Antigen-specific T cells induced by parenteral vaccination remain confined to tissue compartments outside of the lung parenchyma and the airways, and are thus not capable of mediating complete protection against *Mtb* (15, 55). Therefore, a vaccination strategy, which induces and maintains tissue-resident memory T cells and/or circulating T cells capable of rapid influx into the lungs upon pathogen re-exposure, may provide robust protection against *Mtb* infection. Parenteral administration of the TB subunit vaccine H56/CAF01 has been shown to induce promising protective efficacy in mice (29) and non-human primates (30). However, the H56/CAF01 vaccine has never been evaluated following airway (i.pulmon.) mucosal immunization. Here, we document the immunogenicity of the H56/CAF01 vaccine following parenteral (i.m.) priming and airway (i.pulmon.) boost immunization, which induces local lung and systemic memory CD4^+^ T cells and IgA responses. We further describe, for the first time, the SPECT-CT imaging-based *in vivo* biodistribution and pharmacokinetics of the H56/CAF01 vaccine following parenteral (s.c.) or airway (i.pulmon.) priming or parenteral prime-airway mucosal boost (s.c.- i.pulmon.) immunization strategy. We observe pronounced differences in the deposition, biodistribution and clearance of H56 protein and CAF01 adjuvant. Our study provides new information on H56/CAF01 mucosal boost immunization-inducible memory CD4^+^ T cells in the lungs and SPECT/CT imaging-based *in vivo* biodistribution of the individual vaccine components, which may assist the development of effective mucosal immunization strategies against pulmonary TB. These results also support our ongoing efforts to develop a thermostable, dry powder-based H56/CAF01 vaccine for i.pulmon. administration (43), and we envisage the potential application of such an inhalable dry powder dosage form in combination with a suitable device for mass vaccination programs against TB. Hence, the safety of airway mucosal vaccination has to be evaluated thoroughly in future studies.

It is well-known that T cells play a crucial role in the pulmonary host defense against many bacterial, viral, and fungal pathogens, and inadequate T-cell immunity may increase the likelihood of pathogen dissemination from the lungs (9). It is also well-known that MHC class II-restricted CD4^+^ T cells producing IFN-γ and TNF-α play important roles in protection against TB in experimental animal models and in humans (56, 57). In many preclinical TB challenge studies, increased CD4^+^ central memory T cells have been associated with enhanced protection (14, 57, 58). Localization of antigen-specific CD4^+^ T cells at the site of infection in the lung parenchyma is of ultimate importance for disease protection after vaccination (52, 56). However, *Mtb* infection greatly interferes with migration of circulating vaccine-induced antigen-specific T cells to the lungs (19), which is correlated with lack of protection (15, 59). Therefore, novel immunization strategies are needed to induce T cells that effectively home back to the lung parenchyma in the airways. In the present study, we introduce an effective immunization protocol for the H56/CAF01 vaccine, which results in induction of strong Th1, Th17, and IgA responses in the airways. We show that Th1 and Th17 cells are induced systemically after airway mucosal boost immunization of parenterally primed H56 antigen in a CAF01 dose-dependent manner, and that local lung mucosal and systemic IgA and IgG responses accompany this. We also show that mucosal immunization induces polyfunctional (IFN-γ^+^TNF-α^+^IL-17^+^) and double positive (IFN-γ^+^TNF-α^+^ and TNF-α^+^IL-17^+^) and TNF-α single-positive CD4^+^ T cells in the lungs and the lung draining LNs (TLNs + MLNs) as well as the spleen. The H56/CAF01 vaccine has previously been shown to preferentially induce accumulation of TNF-α single-positive, double positive (IFN-γ^+^TNF-α^+^ and TNF-α^+^IL-2^+^) and triple-positive (IFN-γ^+^TNF-α^+^IL-17^+^) CD4^+^ T cells in the lungs, which provide protection against an *Mtb* challenge (29). Our results also support previous observations that less differentiated H56-specific T-cells have increased ability to migrate into the lung parenchyma (39, 60). However, we also observe CD4^+^ T cells with an intermediate state of differentiation (IFN-γ^+^TNF-α^+^), as usually seen post-*Mtb* infection (39, 60, 61). The induction of this population following prime-boost immunization could suggest that innate immune factors in the lung microenvironment play an important role for the extent of cell differentiation (62). However, the maintenance of an IFN-γ^+^TNF-α^+^ double positive T-cell population has been associated with enhanced control of mycobacterial growth (56, 57). Similarly, parenteral prime and mucosal boost immunization were shown to induce strong mucosal and systemic immunity (16, 63, 64), accompanied by improved protection in a number of preclinical infectious disease models (65–67). Intranasal boosting of parenterally primed immune responses was associated with improved protection against *Mtb* infection, which correlated with IFN-γ^+^ CD4^+^ and CD8^+^ T cells residing in the airway lumen of the lungs (68). Similarly, respiratory mucosal boosting of parenteral immunization resulted in improved protection against *Mtb* infection, and it was accompanied by antigen-specific T cell responses in the lungs (13, 69). Together with our data, these findings suggest that parenteral prime and mucosal boost immunization is a potentially effective strategy for inducing lung-resident CD4^+^ T cells, which can subsequently provide an improved protection against an *Mtb* challenge. We are currently testing this strategy in an *Mtb* challenge model.

Almost all licensed vaccines against infectious diseases induce antibodies, which are correlated with disease protection (70). Antibody-mediated protective immunity is mediated by mucosal IgA, which prevents pathogen uptake across the epithelial barrier, and by serum IgG, which prevents further pathogen transmission *via* the blood (71). However, the role of B cells and their production of antibodies in the immune response to *Mtb* infection remains elusive (72–74). Recently, there is growing evidence that *Mtb*-specific antibodies may contribute to prevention of TB (75, 76), and one study reported that antibodies recovered from healthcare workers provided moderate protection against *Mtb* in mice (76). Furthermore, a number of experimental studies have shown a protective effect of antibodies against *Mtb* surface glycolipids (77, 78) and recombinant antigens (79, 80). The induction of antigen-specific IgA and IgG responses following prime-boost immunization in our study strengthens these findings. Ideally, vaccine-induced mucosal IgA antibodies present at the natural portal of entry in the lungs, which are capable of fast neutralization of *Mtb* following exposure, would be the optimal preventive strategy against TB. In line with this, passive protection by mucosally administered human IgA antibodies against *Mtb* infection in the lungs of mice has been reported (81, 82). Recently, vaccine-induced pulmonary secretory IgA has been associated with immunological protection against TB in mice (17, 83). Given the fact that antibodies are protective against many intracellular infections, further studies are required to verify the functional differences in antibodies to *Mtb* and the precise role of mucosal antibodies in the immunological protection against TB (84).

In this study, we successfully radiolabeled CAF01 liposomes with a lipophilic chelator, and developed an ^111^In-DTPA-CAF01 complex with high radiolabeling efficiency and purity. We also describe the successful design of ^111^In-DTPA-H56 and ^67^Ga-NOTA-H56 complexes, respectively, with high radiopurity and radiolabeling efficiency. Radiolabeling of both H56 and CAF01 was not only easy and reproducible, but did also result in preserved size and integrity of both protein and liposomes. The ^111^In and ^67^Ga radionuclides were selected due to their relatively long half-life (^111^I *n* = 2.81 and ^67^Ga = 3.26 days), their high photon energy, their ready and daily availability as cyclotron-produced radionuclides, and the possibility for clinical translation. Using SPECT-CT imaging, we confirmed our previous studies showing that CAF01 forms a depot at the site of injection (48). First, we show that ^111^In-CAF01 with surface-adsorbed cold H56 and ^111^In-H56 adsorbed onto cold CAF01 remain at the site of injection for up to 6 days post-s.c. injection, and we confirmed the observation through *ex vivo* biodistribution. Using the tracer molecule ^3^H-DPPC with DDA and TDB and ^125^I-labeled Ag85B-ESAT6 protein (so-called H1), it was shown previously that CAF01 forms a depot when injected i.m. or s.c. and promotes antigen retention at the site of injection (48). Moreover, this depot effect was correlated with the synchronization of DC uptake of antigen and activation by CAF01, which is an important element for the Th1/Th17 adjuvanticity of CAF01 (85, 86). However, in our study, there was a clear difference in the biodistribution of H56 and CAF01 following s.c. injection; H56 was cleared faster than CAF01 and was detected in the kidneys and the bladder already 6 h post-administration. Following pulmonary administration of radiolabeled H56 and CAF01, respectively, we generally observed rapid accumulation of radioactivity in the lungs and the bladder within the first hour post-administration. The observed activity in the trachea is caused by deposition of very small amounts of activity from the tip of the MicroSprayer® needle during i.pulmon. administration. At later time-points, the amount of radioactive H56 in the lungs, although lower, was detectable until 24 h, as compared to radioactive CAF01, which was observed until 96 h. For both H56 and CAF01, radioactivity was observed in the stomach and the intestines from 6 to 24 h post-injection, which could be due to cough reflux during the withdrawal of the MicroSprayer® needle from the trachea, or clearance of the dispersion from the trachea and subsequent swallowing, as previously reported (47). Nevertheless, the applied i.pulmon. administration method enables a rather uniform distribution of the aerosolized vaccine into both lung lobes as observed by SPECT imaging. This is further supported by the fact that extrapulmonary distribution following aerosolization did not influence the overall vaccine immunogenicity, and it was not associated with any apparent side effects or systemic toxicity during the study period (up to a maximum of 6 weeks).

Induction of cell-mediated immunity by vaccination is challenging. It is believed that repeated administration of the same vaccine (homologous boosting) is effective for increasing humoral but not cellular immune responses, while heterologous prime-boost immunization induces strong humoral and cellular immune responses (87, 88). However, the use of the homologous parenteral prime-mucosal boost immunization schedule has been shown to induce simultaneous robust local mucosal and systemic protective cellular and humoral immunity against mucosal pathogens, e.g., *Mtb* and HIV (13, 63, 69). Our data show that homologous parenteral priming followed by airway boosting with H56/CAF01 elicits strong antigen-specific CD4^+^ T cell responses, both in the spleen and the lungs, and IgA responses in both serum and lungs, as compared to parenteral homologous prime-boost immunizations. Recently, it was reported that the administration route used for priming and boosting of the H56/CAF01 vaccine is important for improving and directing the vaccine-induced immune responses using either the homologous or heterologous prime-boost combinations (22). Largely, the enhanced immunity following prime-boost homologous or heterologous immunization to the target antigen is reflected predominantly by cellular events, e.g., an increased number of antigen-specific T cells, enrichment of high-avidity T cells, and subsequent increased protective efficacy against a pathogen challenge (89). Having demonstrated significantly higher CD4^+^ T-cell- and antibody responses for homologous prime-boost immunization, we evaluated the biodistribution and pharmacokinetics of the H56/CAF01 vaccine by SPECT/CT imaging to compare pulmonary uptake and distribution between airway (i.pulmon.) prime vs. parenteral (s.c.) prime—airway (i.pulmon.) mucosal boost immunization strategies. However, the vaccine biodistribution in airway-boosted animals that were primed s.c. with the homologous vaccine was not significantly different from the biodistribution in s.c.- primed only animals. Since most of the immunological events are taking place at the cellular level initially in the lung mucosa and draining lymph nodes followed by systemic circulation, whole-body SPECT/CT imaging cannot be used to differentiate the cellular events during prime vs. prime-boost immunization. Nevertheless, the comparable vaccine biodistribution profiles upon homologous prime and prime-boost immunization underlines the reproducibility of our radiolabeling results and emphasizes the usability of the SPECT/CT imaging-based approach for quantification of the biodistribution of subunit vaccines. Our novel data represent dual-isotope radiolabeling and preclinical non-invasive and longitudinal SPECT/CT imaging of the H56/CAF01 vaccine as a readily translatable strategy, which can be integrated into a clinical workflow. In addition, this novel radiolabeling platform can be used to identify image-derived biomarkers, which could be used to image vaccine-induced immune response, where imaging of sites such as lungs, LNs and spleen can provide additional information about vaccine-induced immune response as well as safety and efficacy. Interestingly, a recent study reported a PET imaging-derived biomarker that can be used to image activated T cells to predict tumor responses to *in situ* vaccination (90). Future studies should include devising novel immuno-SPECT/CT strategies for the identification of H56/CAF01 vaccine-induced activated T cells for differentiating prime vs. prime-boost immunizations and corresponding vaccine efficacy.

From our data we can conclude that strong IgA antibody and polyfunctional Th1 and Th17 cell responses are induced in the lung mucosa and the systemic circulation upon parenteral (i.m.) priming combined with airway (i.pulmon.) mucosal boost immunization with the TB subunit vaccine H56/CAF01, as compared to parenteral (i.m.) priming combined with parenteral (i.m.) boost immunization. These data demonstrate that parenteral priming followed by airway mucosal boosting with the H56/CAF01 vaccine is a novel immunization strategy for improving vaccine immunogenicity and directing the trafficking of antigen-specific CD4^+^ T cells to the lungs. These results warrants further preclinical and clinical development of H56/CAF01 as an inhalable and self-administrable aerosol vaccine. We conclude that there are very pronounced differences in the pharmacokinetics of H56 and CAF01 based on dual isotope (^111^In/^67^Ga)-based SPECT/CT imaging of the vaccine biodistribution. Our results also suggest a comparable biodistribution profile of the H56/CAF01 vaccine following airway (i.pulmon.) prime and parenteral prime (s.c.)—airway (i.pulmon.) mucosal boost immunization, respectively. We believe that immuno-SPECT/CT strategies can be developed, based on this novel radiolabeling platform, for imaging of H56/CAF01 vaccine-induced activated T cells at specific effector sites, e.g., the lungs. Overall, our findings may hold considerable implications for the rational design of effective mucosal immunization strategies against TB.

## Author contributions

AT, DC, UH, and CF designed the study. AT, CR-R, KS, FR, TE, and ZN performed the laboratory work and analyzed the data. AT, DC, UH, and CF interpreted the data. AT, CR-R, UH, and CF drafted the manuscript. AT, CR-R, KS, PA, DC, UH, and CF provided scientific input throughout the study period and draft of the manuscript.

### Conflict of interest statement

PA and DC are employed by Statens Serum Institut, a non-profit government research facility, of which the CAF adjuvants and H56 are proprietary products. The remaining authors declare that the research was conducted in the absence of any commercial or financial relationships that could be construed as a potential conflict of interest.

## Abbreviations

BCG: Bacillus Calmette–Guérin
CAF01: cationic adjuvant formulation 01
CT: computed tomography
DC: dendritic cell
DDA: dimethyldioctadecylammonium
DTPA: diethylenetriamine pentaacetic acid
Ga: gallium
ILN: inguinal lymph node
In: indium
i. pulmon.: intrapulmonary
ITLC: instant thin layer chromatography
MBq: megabecquerels
MLN: mediastinal lymph node
Mtb: *Mycobacterium tuberculosis*
NOTA: 1,4,7-triazacyclononane-1,4,7-triacetic acid
PDI: polydispersity index
PLN: popliteal lymph node
Rf: retention factor
SPECT: single-photon emission computed tomography
SUV: standardized uptake value
TB: tuberculosis
TDB: trehalose-6,6'-dibehenate
TLN: tracheobronchial lymph node
VOI: volume of interest.
