# Supplementary figures and images for: Dual-Isotope SPECT/CT Imaging of the Tuberculosis Subunit Vaccine H56/CAF01: Induction of Strong Systemic and Mucosal IgA and T-Cell Responses in Mice Upon Subcutaneous Prime and Intrapulmonary Boost Immunization
Source: Front Immunol. 2018 Nov 30;9:2825. doi: 10.3389/fimmu.2018.02825 (PMC6284049; doi:10.3389/fimmu.2018.02825)

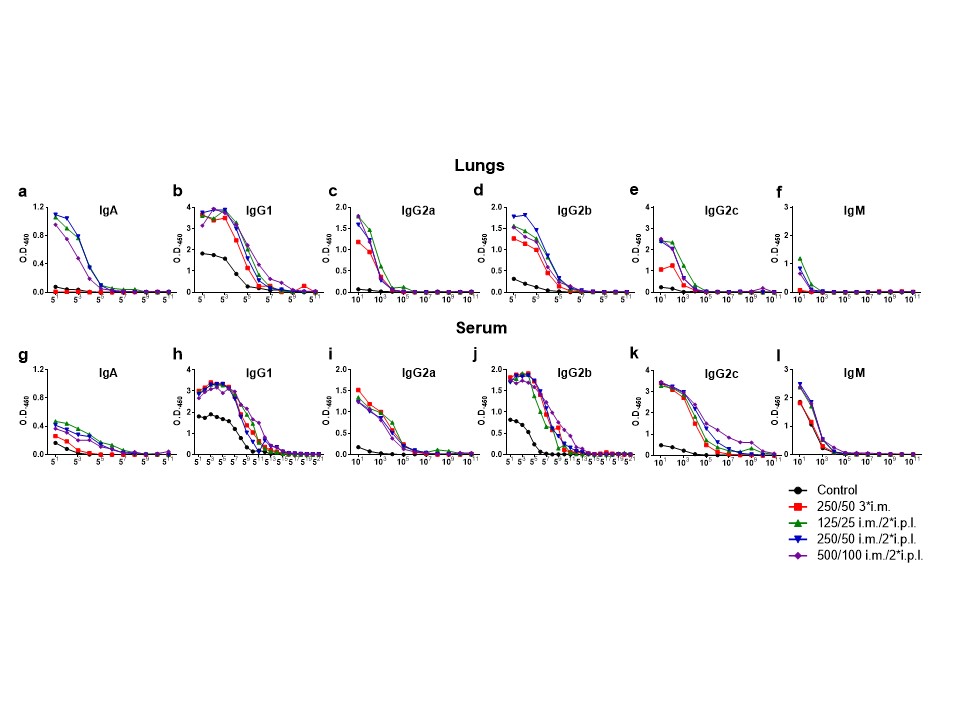

Supplement: Supplementary file 1 [file Image_1.JPEG]

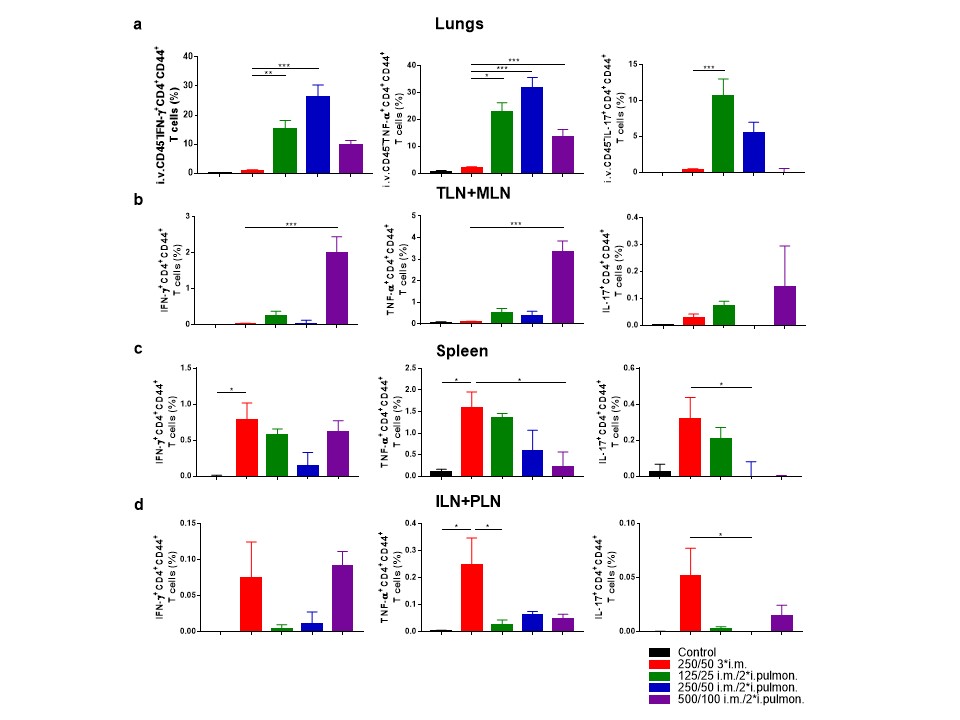

Supplement: Supplementary file 2 [file Image_2.JPEG]

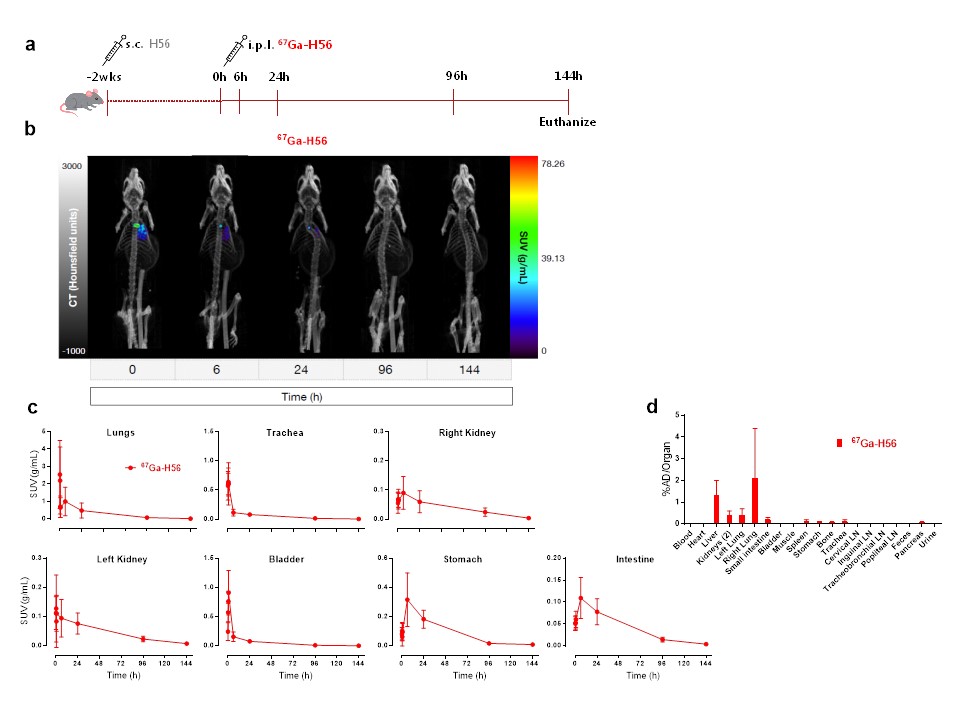

Supplement: Supplementary file 3 [file Image_3.JPEG]

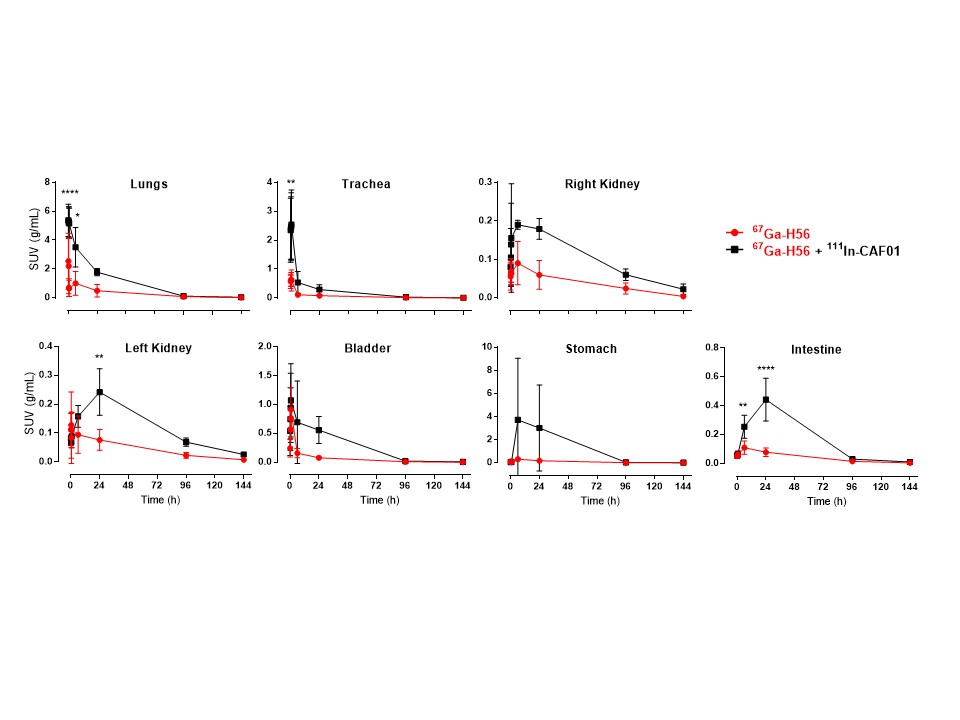

Supplement: Supplementary file 4 [file Image_4.JPEG]
