# Supplementary material for: Dual-Isotope SPECT/CT Imaging of the Tuberculosis Subunit Vaccine H56/CAF01: Induction of Strong Systemic and Mucosal IgA and T-Cell Responses in Mice Upon Subcutaneous Prime and Intrapulmonary Boost Immunization
Source: Front Immunol. 2018 Nov 30;9:2825. doi: 10.3389/fimmu.2018.02825 (PMC6284049; doi:10.3389/fimmu.2018.02825)
Supplement: Supplementary file 5 [file Data_Sheet_1.docx]

**Supplementary information**

**Materials and Methods**

*SPECT/CT image reconstruction*

For the SPECT images, we used 16 subsets, 10 iterations and an isotropic 0.4 mm voxel grid. The images were decay-corrected, and after CT registration, attenuation corrected. For visual representation, the reconstructed volumes of the SPECT scans were post-filtered using a 3D Gaussian filter. The CT scans were acquired using a tube setting of 45 kV and 615 µA. In total, two frames of 180 projections over 360 degrees were acquired in step and shoot rotation mode. The acquired projection data was reconstructed using SkyScan NRecon software (Bruker microCT, Kontich, Belgium) to generate a 3D CT image of 0.169 mm^3^ voxel size. Volumes of interest (VOIs) were manually defined using AMIDE (v.1.0.5, Stanford University, Stanford, CA, USA) to determine the time activity pattern per target organ. Thus, the delineated regions were lungs, site of injection, trachea, right and left kidney, bladder, stomach and intestines. The precise positions of the VOIs were adjusted using the registered CT image for each scan. In order to relate the scanner units (counts/voxel) to radioactivity concentration, a calibration factor was determined by scanning a source with a known concentration of ^111^In and ^67^Ga. The mean activity in each VOI (counts/pixel) was multiplied by the calibration factor to calculate the activity concentration in each organ (MBq/mL). Results are expressed as standardized uptake values (SUVs) to normalize the reached organ concentrations by taking into account the individual body weight and the administered dose according to the following equation (Equation 1):

SUV (g/mL) = [activity concentration in VOI (MBq/mL)/(administered dose (MBq) x body weight (g))] (Equation 1)

All SPECT images were decay-corrected to the time of administration. Mice were euthanized for *ex vivo* quantification of the biodistribution, and the radioactivity in the organs was determined by γ-counting.

**Supplementary Table S1.** Secondary antibodies for the detection of antibody production in lung supernatants and serum

| **Isotype** | **Antibody** | **Supplier** | **Dilution** |
| --- | --- | --- | --- |
| IgA | HRP-conjugated goat anti-mouse IgA | Invitrogen, Hvidovre, Denmark | 1:5,000 |
| IgG1 | HRP-conjugated goat anti-mouse IgG1 | Southern Biotech, Birmingham, AL, USA | 1:20,000 |
| IgG2a | HRP-conjugated rabbit anti-mouse IgG2a | Invitrogen, Hvidovre, Denmark | 1:5,000 |
| IgG2b | HRP-conjugated rabbit anti-mouse IgG2b | Invitrogen, Hvidovre, Denmark | 1:5,000 |
| IgG2c | HRP-conjugated rabbit anti-mouse IgG2c | Southern Biotech, Birmingham, AL, USA | 1:5,000 |
| IgM | HRP-conjugated goat anti-mouse IgM | Southern Biotech, Birmingham, AL, USA | 1:5,000 |

**Results**

**Supplementary Figure S1.** Antigen-specific antibody responses in lung supernatants and serum measured by ELISA. Lung supernatants and serum samples were isolated two weeks after the last booster immunization (week 6 of the study). H56-specific production of IgA, IgG1, IgG2a, IgG2b, IgG2c, and IgM was measured in the lung supernatant (a-f) and serum (g-l) by ELISA.

**Supplementary Figure S2.** Percentage of IFN-γ^+^CD4^+^CD44^+^ or TNF-α^+^CD4^+^CD44^+^ or IL-17^+^CD4^+^CD44^+^ T cells by intracellular flow cytometry. Lung cells were examined for labeling with i.v.-injected, FITC-conjugated anti-CD45.2 mAb. IFN-γ^+^ or TNF-α^+^ or IL-17 producing i.v.CD45^-^CD4^+^CD44^+^ T cells were then identified by gating of lung cells as described earlier (a). Similarly, the percentages of IFN-γ^+^ or TNF-α^+^ or IL-17 producing CD4^+^CD44^+^ T cells were identified in the lung-draining LNs (TLN + MLN) (b), spleen (c), and LNs draining the site of i.m. injection (ILN + PLN) (d). Statistical analysis: one-way ANOVA and Tukey’s post-test. Bars represent mean values ± s.e.m., n = 6. ****p* < 0.001.

**Supplementary Figure S3.** Unadjuvanted H56 is rapidly cleared. (a) Experimental scheme: Mice were prime-immunized s.c. with 5 µg H56. At week 2, animals were boost-immunized *via* the intrapulmonary (i.pulmon.) route with 10 µg ^67^Ga-H56. Animals were imaged by dynamic whole-body SPECT/CT scan for the initial 40 min (10 min/frame) and after that static 40 min scans at 6 and 24 h, 60 min scan at 96 h and 90 min scan at 144 h were conducted. Animals were euthanized on day 6 after immunization for *ex vivo* biodistribution using a gamma counter. (b) Representative SPECT/CT images of a mouse prime-immunized s.c. with cold H56 and boost-immunized i.pulmon. with ^67^Ga-H56 and imaged over 144 h post-immunization. (c) Organ SUVs (g/mL) were calculated from dynamic and static SPECT/CT images of the cold H56 prime-immunized and hot ^67^Ga-H56 boost-immunized animals over 144 h post-immunization (n = 3). (d) *Ex vivo* organ biodistribution (% administered dose (AD)/organ] of the cold H56 prime-immunized and hot ^67^Ga-H56 boost-immunized animals on day 6 (144 h) post-immunization (n = 3).

**Supplementary Figure S4.** H56 has a distinct biodistribution profile when administered in the presence or absence of CAF01 liposomes. The SUVs (g/mL) were compared in the animals that were prime-immunized s.c. with 5 µg cold H56 or 5 µg cold H56 adjuvanted with cold CAF01 (250/50 µg DDA/TDB). At week 2, animals were boost-immunized *via* the intrapulmonary (i.pulmon.) route with 10 µg ^67^Ga-H56 or 10 µg ^67^Ga-H56 adjuvanted with ^111^In-CAF01 (125/25 µg DDA/TDB). Statistical analysis: two-way ANOVA and Sidak’s post-test. Data represent mean values ± s.d., n = 3. **p* < 0.05, ***p* < 0.01 and *****p* < 0.0001.
